# A Preclinical Multifunctional Capsule Robot with Unified Diagnosis and Targeted Thermal Therapy for Gastrointestinal Diseases

**DOI:** 10.1002/advs.77508

**Published:** 2026-09-06

**Authors:** Heng Zhang, Yichong Sun, Xiaoting Zhang, Chi‐Kwan Lee, Zheng Li, Yehui Li, Huarong Chen, Paul D. Mitcheson

**Affiliations:** ^1^ Department of Electrical and Computer Engineering The University of Hong Kong Pokfulam Hong Kong China; ^2^ Department of Surgery The Chinese University of Hong Kong Hong Kong China; ^3^ Department of Anaesthesia and Intensive Care The Chinese University of Hong Kong Hong Kong China; ^4^ School of Electrical and Data Engineering The University of Technology Sydney Sydney (Ultimo) New South Wales Australia; ^5^ School of Computer Science and Engineering Sun Yat‐sen University Guangzhou China; ^6^ Department of Electrical and Electronic Engineering Imperial College London London United Kingdom

**Keywords:** magnetic actuation, multifunctional capsule robot, thermal ablation, wireless power transfer

## Abstract

Capsule endoscopes play an essential role in visualizing the gastrointestinal tract and identifying pathological lesions, but existing systems are still largely limited to passive visualization without therapeutic capability. Here, we present a proof‐of‐concept multifunctional capsule robot (MCR) integrating diagnostic imaging, magnetic navigation, wireless power transfer, and targeted thermotherapy. In the ex vivo environment, the MCR relies on magnetic navigation and control to position itself at the target site. This enables site‐specific thermal ablation, where controlled high temperatures are applied within the tissue to achieve substantial cellular destruction. These results support the feasibility of the prototype's intended functions. Through a series of in vitro cellular experiments, the MCR further demonstrated controllable temperature regulation, achieving up to 89% cell mortality under 70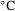 heating for 120 s. The findings demonstrate the feasibility of the proposed approach at a proof‐of‐principle level. The experiments validate the system‐level feasibility of integrating diagnostic imaging, targeted treatment, and wireless charging within one capsule. At this stage, the platform remains a preclinical prototype and is not ready for clinical translation; further engineering and preclinical validation are required. Nonetheless, this MCR architecture provides a preliminary framework that may support future improvements in minimally invasive gastrointestinal therapeutic systems.

## Graphical abstract

A proof‐of‐concept multifunctional capsule robot integrates diagnostic imaging, magnetic navigation, wireless power transfer, and targeted thermotherapy for gastrointestinal applications. Magnetically guided positioning and stable wireless power enable localized thermal ablation, achieving controlled heating and high cell mortality in vitro. This platform demonstrates system‐level feasibility for future minimally invasive diagnostic and therapeutic capsule systems.

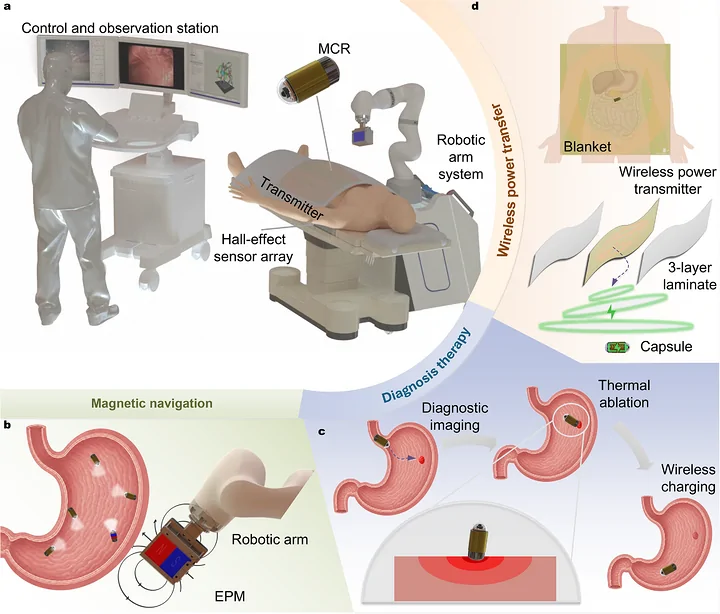

## 1 Introduction

Colorectal and stomach cancers ranked third and fifth, respectively, among all malignancies in terms of global incidence in 2022, posing a significant threat to human health and together accounting for more than 1.5 million deaths worldwide [1]. These substantial disease burdens motivate the continued development of technologies for earlier detection and localized intervention. Flexible endoscopy has long been regarded as the gold standard in clinical gastroenterology; however, it requires invasive procedures that may cause patient discomfort and carry risks of complications [2, 3]. In contrast, wireless capsule endoscopy (WCE), introduced in 2000 [4], has expanded gastrointestinal diagnostics by enabling non‐invasive imaging of the digestive tract [5]. A typical capsule endoscope contains several major components, including housing, optical window, LED array, optical lens, CMOS image sensor, RF transmitter, antenna, and power supply [6]. Swallowed by the patient, the capsule endoscope relies on natural peristalsis of the GI tract for movement and captures a large number of images for examination [6, 7]. Nevertheless, the passive and uncontrollable motion of the capsule endoscope confines its role to imaging‐based screening. Moreover, there is currently no solution for conducting a complete non‐invasive diagnosis and therapy of the gastrointestinal tract in a single medical procedure, a long‐standing aspiration in gastrointestinal medicine.

Recent advances in microrobotics have enabled diverse actuation approaches for capsule robots, including hydraulics [8], magnetics [9, 10, 11, 12, 13, 14], electromechanics [15, 16, 17, 18, 19], and shape memory alloys [20, 21, 22, 23]. These developments transform the conventional passive WCE into an active device, enabling advanced features such as tissue sampling, targeted drug delivery, and thermal ablation of precancerous lesions. Among these driven mechanisms, magnetic actuation stands out for its non‐contact operation, precise manipulability, and device miniaturization [24, 25, 26], as reported in recent studies on capsule endoscopy [27, 28]. These studies have supported the continued development of magnetically driven capsules for biomedical applications. Despite these advances, existing magnetic capsule systems still face two key challenges: (1) most systems primarily serve diagnostic imaging purposes without integrated therapeutic functions; and (2) the power requirements for combined diagnostic and therapeutic operation exceed the capacity of conventional capsule batteries, while the small form factor limits onboard energy storage. It is difficult to apply capsule robots to long‐duration diagnosis and therapy in the GI tract with, a long duration ranging from approximately 24–72 h [29, 30]. These limitations have prevented the realization of a comprehensive capsule platform capable of simultaneous high‐definition imaging, active navigation, localization, and targeted therapy.

To address these limitations of existing capsule endoscope systems, this article presents a proof‐of‐concept multifunctional capsule robot (MCR) that integrates diagnostic imaging and targeted therapy, offering a possible direction for addressing the limitations of current passive capsule endoscopy systems. The developed capsule robot system combines real‐time high‐definition video transmission, active motion control through external magnetic manipulation, six‐degree‐of‐freedom localization using a Hall‐effect sensor array and an inertial measurement unit, as well as targeted thermal ablation capabilities. In addition, the MCR incorporates wireless charging to overcome energy limitations, enabling prolonged operation throughout the complete GI transit time. Through a series of ex vivo and in vitro experiments, the MCR demonstrated precise temperature control and site‐specific thermal ablation, achieving up to 89% cancer cell mortality under 70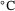 heating for 120 s. To the best of our knowledge, this work represents an early proof‐of‐concept demonstration of a capsule robot that integrates diagnostic imaging with localized thermal ablation capability within a single ingestible platform.

## 2 System Overview

**FIGURE 1:**
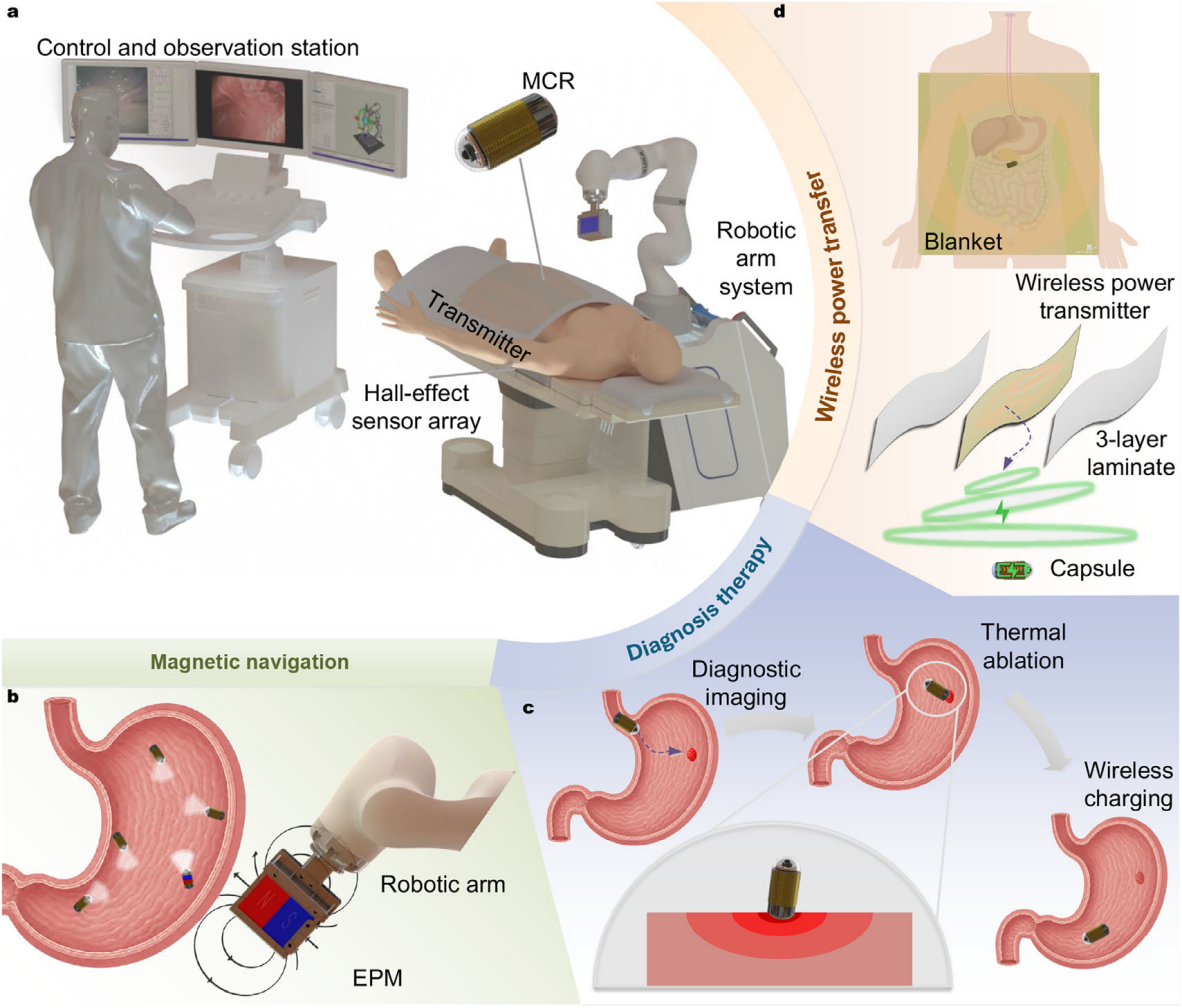
Overview of the multifunctional capsule robotic system and workflow. (a) Clinical setup. A patient lying on the examination table, covered by a blanket embedded with WPT capability. A medical practitioner controls the robotic arm from the control station. The localization and orientation of the capsule are determined using a Hall‐effect sensor array beneath the patient and an inertial measurement unit (IMU) in the capsule. (b) Details of the robotic arm's end‐effector are illustrated. It holds a permanent magnet that anchors the capsule to the inner wall of the patient's stomach through magnetic attraction. As the permanent magnet moves or rotates, it alters the magnetic force and torque on the magnet inside the capsule, enabling translational and rotational motions. (c) A thermal ablation treatment is depicted, where a lesion is identified in the stomach. The robotic arm generates a dragging force to move the capsule closer to the lesion, allowing the capsule to apply localized heat to ablate the abnormal tissue. d, Video transmission and heat therapy quickly drain the capsule's battery. A wireless power transmitter generates a high‐frequency electromagnetic field to charge the capsule's battery.

Figure 1 provides an overview of the proof‐of‐concept multifunctional capsule robotic system for gastrointestinal imaging, active navigation, wireless power transfer, and localized thermal ablation. The system consists of five modules: a control and observation station, a robotic arm, a multifunctional capsule robot, a localization Hall‐effect sensor array, and a wireless power transfer module (Figure 1a). In operation, the patient swallows the MCR and lies on the examination table. At the control station, a medical practitioner manipulates the robotic arm to adjust the capsule's location and orientation within the patient's GI tract. The MCR contains a permanent magnet that aligns with the external magnetic field generated by a magnet on the robotic arm's end‐effector (Figure 1b). The MCR maintains anchoring and its orientation by using a continuous and substantial magnetic compression force generated between the external permanent magnet (EPM) and the internal MCR magnet. A strong vertical compression force against the mucosal wall is created acting as a virtual mechanical anchor. It effectively prevents the device from being displaced by lateral fluid disturbances. The MCR steering operation requires altering the EPM magnetic dipole to control the tilting angle, while simultaneously adjusting the gap distance between the EPM and MCR magnet in order to maintain a constant vertical compression force for anchoring. The same magnetic‐robotic control system has been introduced in [31], and the control accuracy has been well evaluated. This setup allows for control of the capsule's position and orientation. The MCR provides enhanced diagnostic flexibility compared to conventional capsule endoscopy, which relies entirely on natural bowel peristalsis and gravity to travel through the gastrointestinal (GI) tract.

Using high‐definition video transmitted from the capsule, precancerous lesions and polyps can be identified, and therapeutic procedures, such as thermal ablation, can be performed. The MCR is equipped with a heating element that induces thermal injury to the targeted tissue (Figure 1c). High‐definition video transmission and thermal ablation consume substantial energy and can rapidly deplete the onboard battery. To address this energy demand, a wireless power transfer (WPT) system was developed (Figure 1d). The wireless power transmitter, made from flexible materials, is embedded in a blanket covering the patient. This transmitter consists of multiple flexible coils that generate a three‐dimensional high‐frequency magnetic field. The receiving coil in the capsule harvests and converts this magnetic field into electricity, which is stored in the internal battery.

## 3 MCR Design and Characterization

### 3.1 MCR Design

**FIGURE 2:**
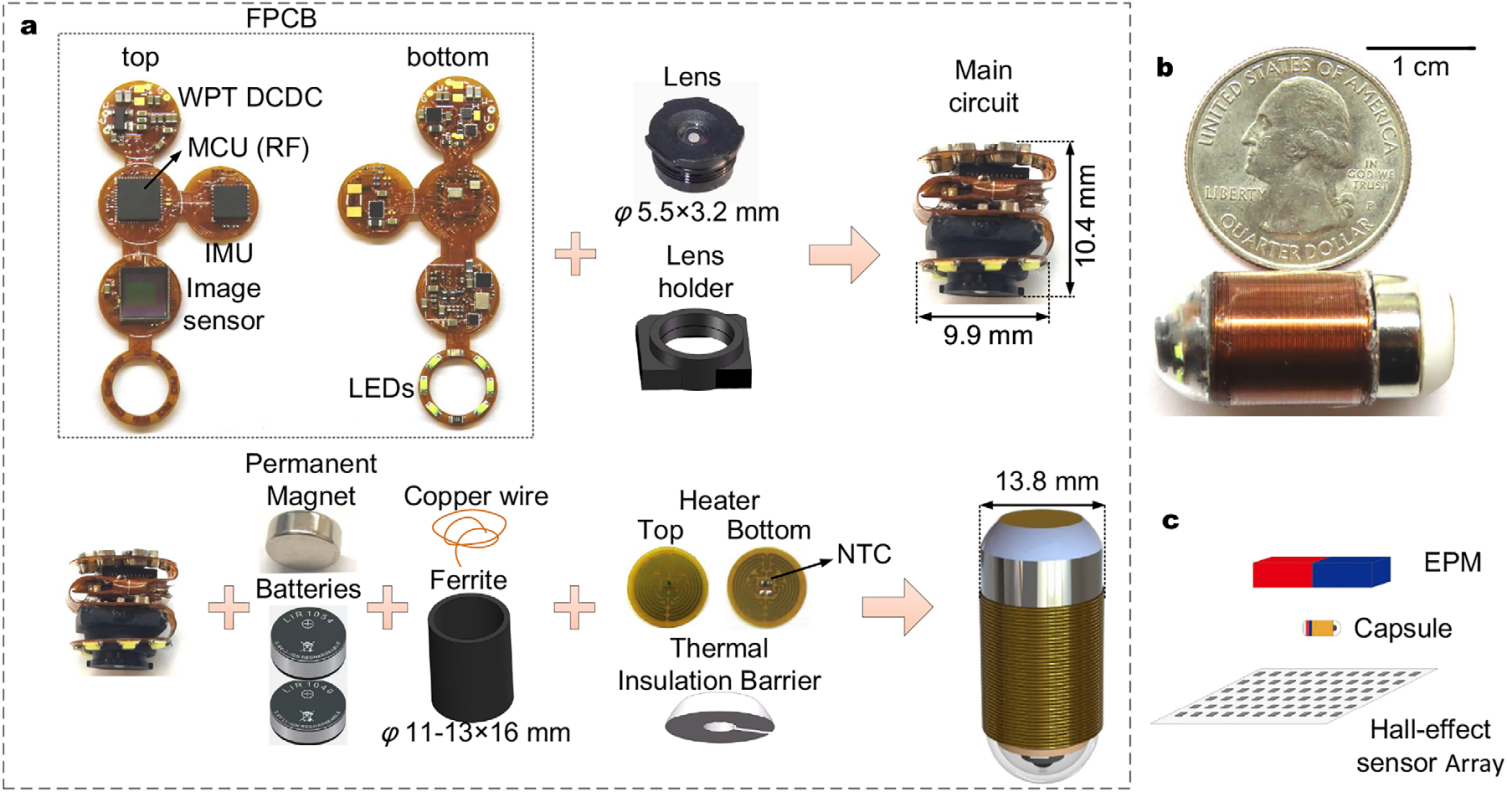
Fabrication, architecture, and system diagrams of the MCR. (a) The entire fabrication process of the MCR, which consists of an FPCB, a camera, a permanent magnet, batteries, a ferrite core, and a heating element. (b) A size comparison of the MCR and a quarter‐dollar US coin is shown. (c) The key components of MCR localization and magnetic field control. The MCR position and orientation are determined by analyzing the magnetic field distribution measured by the Hall‐effect sensor array.

The MCR was developed to achieve high‐definition and high‐frame‐rate video acquisition while integrating diagnostic and therapeutic capabilities. Unlike conventional capsule endoscopes that typically operate at 2 FPS and 512 × 512 resolution, the MCR achieves up to 50 frames per second (FPS) at a resolution of 160 × 120 pixels and supports 3 FPS at the maximum resolution of 1600 × 1200 pixels, enabling real‐time adjustment according to clinical needs. This flexibility allows both comprehensive visualization and targeted observation of lesions. Beyond imaging, the MCR incorporates a thermal ablation module capable of treating detected lesions immediately after identification, thus bridging diagnosis and therapy within a single capsule. The MCR also integrates wireless data transmission (up to 2 Mbps), six‐degree‐of‐freedom (6D) pose estimation, and wireless charging, which together support its therapeutic intervention capabilities. As illustrated in Figure 2, the MCR features a compact, integrated structure housing a high‐resolution camera with LED illumination, a microcontroller, power‐management circuitry, a permanent magnet, batteries, and a wireless‐charging unit. The EPM enables external pose control and facilitates localization through a Hall‐effect sensor array (Figure 2c).

### 3.2 Magnetic Localization

**FIGURE 3:**
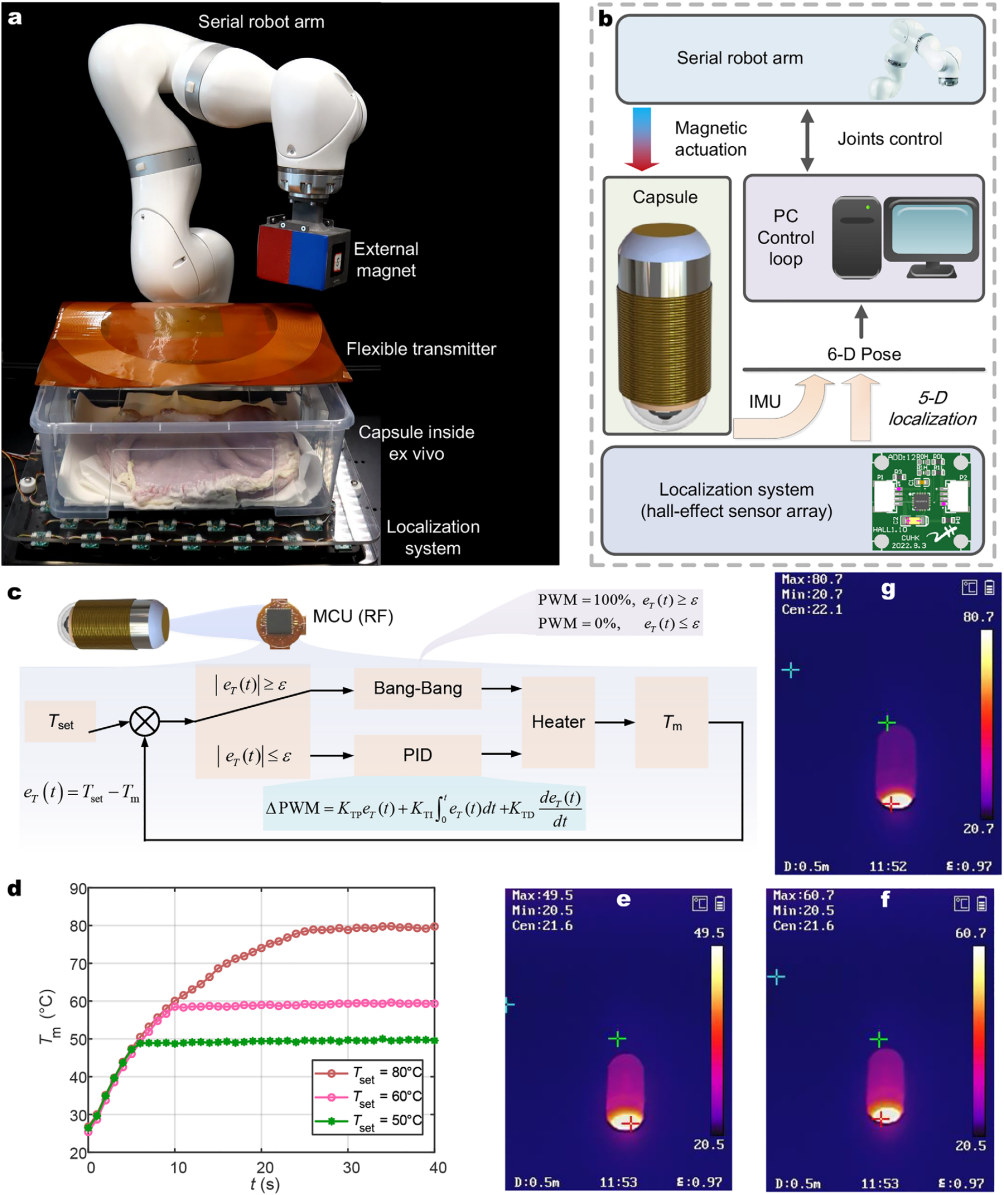
Ex vivo experiment and performance measurements. (a) Photograph of the experimental setup used for the ex vivo validation. (b) Block diagram of the overall system framework and positioning‐drive framework. (c) Temperature control diagram of the MCR. d, Measured temperature profiles under different target temperatures. (e, f, and g) Thermal distribution images acquired using an infrared thermal camera during MCR‐induced heating process at temperatures of 50 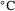, 60 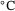, and 80 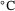, respectively.

The magnetic localization and actuation system was developed to enable control of the MCR position and orientation in an ex vivo environment. Figure 3a shows the hardware, and Figure 3b illustrates the operational framework. The actuation system is a robotic arm, which is articulated with high degrees of freedom. It enables position and orientation control to manipulate the external permanent magnet on the robotic arm's end‐effector. The robotic arm dynamically changes the position and orientation of the EPM, thereby modulating the magnetic field to guide the MCR within the GI tract. The foundation of the platform is a localization system, which consists of a Hall‐effect sensor array to estimate the position and orientation of the MCR. Orientation feedback from the MCR's inertial measurement unit (IMU) is fused with the Hall‐sensor measurements through a sensor‐fusion algorithm, providing real‐time estimates of the MCR's six‐degree‐of‐freedom pose, including the x, y, and z positions and the roll, pitch, and yaw orientations [31]. A computer system at the control station oversees the entire operation. It receives position and orientation data from the localization system and the MCR. Computer algorithms integrate and analyze these data, transforming them into useful information for the medical practitioner to manipulate the robotic arm. The control station also translates operator input, such as movements of the handheld controller, into joint‐level commands for the robotic arm, thereby enabling closed‐loop control during system operation.

### 3.3 Thermal Ablation

Thermal ablation utilizes localized heat to produce controlled thermal ablation in the treated tissue, offering a minimally invasive therapeutic intervention. This technique leverages the temperature‐dependent nature of cellular viability, wherein specific thermal thresholds induce a range of biological effects, from protein denaturation to irreversible cell damage. The effectiveness of thermal ablation relies on achieving and maintaining target tissue temperatures and is influenced by the applied energy, heat dissipation dynamics, and the tissue's intrinsic thermal sensitivity [32, 33]. Traditional hyperthermia protocols raise tissue temperatures to 42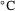 – 45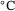 for prolonged periods (30–60 min), resulting in irreversible cellular damage primarily through enzymatic inactivation—one of the hallmarks of thermal injury [34]. When temperatures exceed 60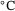, the time required to induce irreversible damage decreases exponentially due to rapid protein denaturation and subsequent cell death. Beyond 100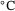, tissue dehydration and water evaporation amplify these effects, while temperatures between 100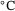 and 300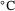 further exacerbate thermal injury through additional mechanisms. At temperatures exceeding 300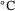, carbonization and char formation occur, which not only limit deeper thermal penetration but also effectively confine tissue damage to the targeted region [35]. These temperature‐dependent mechanisms form the basis of the clinical utility of thermal ablation, particularly in the treatment of tumors and other pathological tissues. The MCR is able to precisely regulate the temperature increase, enabling localized tissue heating that enhances therapeutic precision while minimizing collateral effects on surrounding healthy structures. This capability demonstrates the potential of the system to achieve controlled, site‐specific thermal ablation for minimally invasive therapeutic interventions.

The bottom of the MCR is a heating element with an NTC temperature sensor, facilitating precise temperature regulation by modulating the effective voltage applied to the heating element. The temperature control strategy, illustrated in Figure 3c, employs a two‐stage control algorithm from a bang‐bang controller to a proportional‐integral‐derivative (PID) controller. Based on the temperature error, the control strategy automatically switches between the bang‐bang controller and the PID controller, enabling rapid, accurate, and stable temperature regulation. As illustrated in Figure 3d, three target temperatures, 50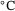, 60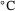, and 80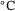, were evaluated.

Under an ambient open‐space temperature of (25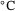), the capsule reaches 50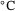 in approximately 6 seconds and 80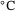 within 26 seconds. The mean absolute temperature errors were 0.29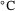, 0.57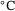, and 0.45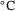, respectively, with corresponding standard deviations of 0.15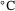, 0.15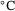, and 0.24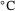. These results show that the heating element can reliably reach thermal thresholds associated with cellular ablation under the tested conditions, supporting its capability to achieve controlled thermal conditions for ablation under experimental settings. The thermal distributions monitored using an infrared thermal imaging camera (Figure 3e–g) confirm that the body temperature of the MCR remains low while the heating module quickly reaches and maintains the designated temperature setpoints, concentrating heat at the bottom of the MCR.

### 3.4 Wireless Charging

To enable wireless charging, the MCR is equipped with a one‐dimensional receiving coil. This approach significantly reduces the space occupied by the wireless charging component compared to traditional multi‐dimensional receiving coils, which capture magnetic fields from various directions. [36, 37, 38, 39, 40]. The wireless power transmitter is fabricated using three‐layer flexible printed circuit board (FPCB). Each layer contains a coil, and the three coils generate alternating magnetic fields along the x, y, and z axes, which are superimposed to create an omnidirectional magnetic field. The magnetic field direction is controlled in real‐time to align with the position and orientation of the MCR, thereby maximizing the charging efficiency.

**FIGURE 4:**
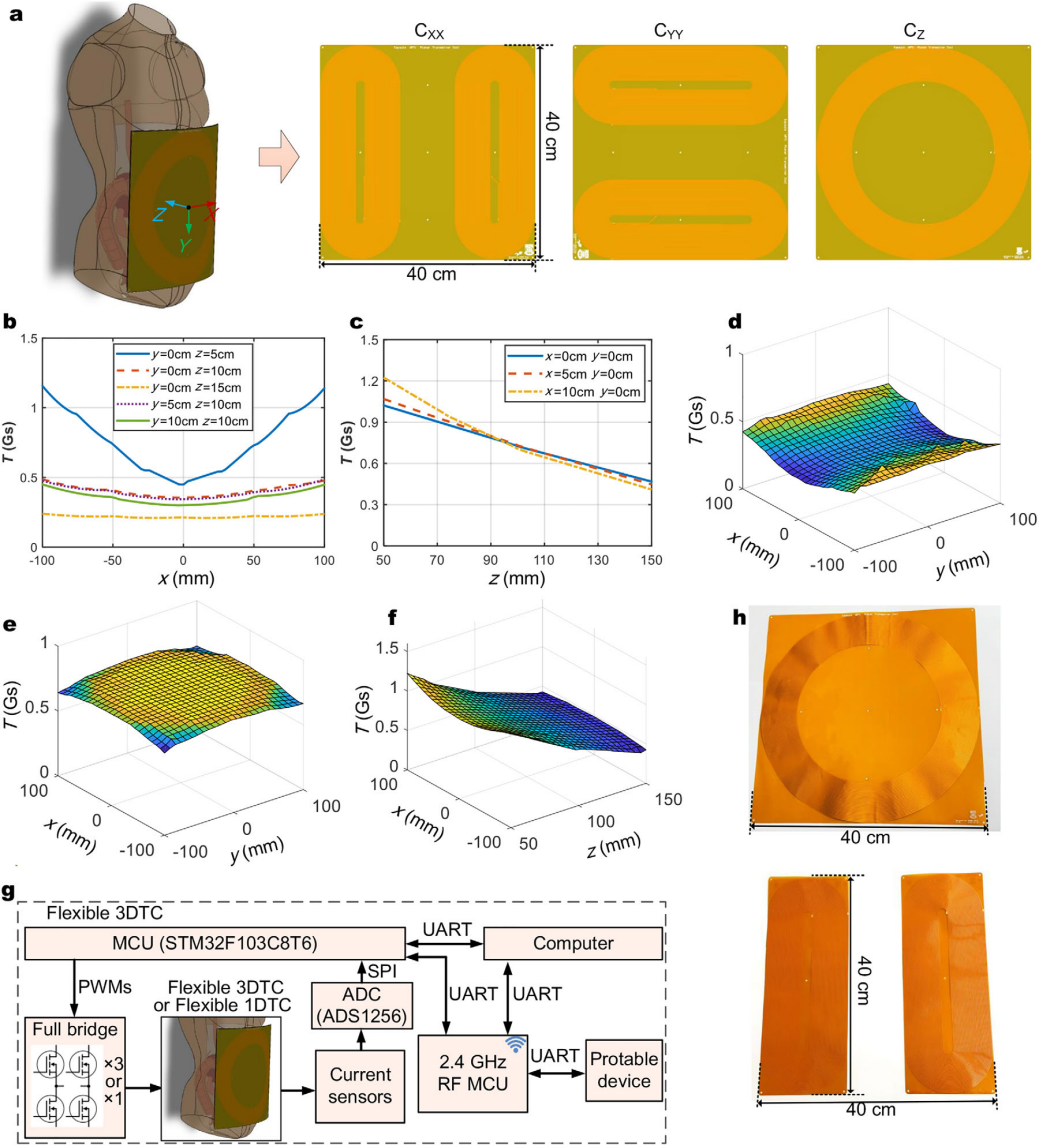
Implementation, measurements and control of wireless charging. (a) Illustration of the flexible transmitting coils, configured to generate magnetic fields along the orthogonal x, y, and z axes for spatially controllable wireless power transmission. (b) Magnetic field profiles are generated by the $C_{\mathrm{XX}}$ coil and are plotted along the x‐axis as the y and z coordinates are varied. (c) Magnetic field profile produced by the $C_{Z}$ coil, plotted along the z axis as the x and y coordinates are varied. (d) Magnetic field profile produced by the $C_{\mathrm{XX}}$ coil plotted in xy plane (z=10cm). (e) Magnetic field profile produced by the $C_{Z}$ coil plotted in xy plane (z=10cm). (f) Magnetic field profile produced by the $C_{Z}$ coil plotted in xz plane (y=10cm). (g) Control block diagram. (h) Photograph of the flexible transmitting coils.

The block diagram of the wireless charging system is shown in Figure 4. Figure 4a illustrates the circuit diagram of the wireless power transmitting coil, which is designed to generate magnetic fields along the x, y, and z axes. Fig. 4b shows the magnetic field distribution produced by the $C_{\mathrm{XX}}$ coil, illustrating the measured field strength along the x‐axis for five different sets of y and z coordinates. The y and z coordinates are constant for each measurement in the data presented. Each set corresponds to a specific combination of y and z values while varying the x‐axis measurement. Similarly, Figure 4c shows the magnetic flux distribution produced by the z coil, presenting the measured field strength along z‐axis for three different sets of x and y coordinates. The spatial distribution of the magnetic field is further depicted in Figure 4d,e,f, which visualize the magnetic field generated by the $C_{\mathrm{XX}}$ coil, $C_{\mathrm{YY}}$ coil, and $C_{Z}$ coil on the xy‐plane at z=100 mm, the xy‐plane at z=100 mm, and the xz‐plane at y=100 mm, respectively. Figure 4g provides a comprehensive control block diagram of the wireless charging system. Figure 4h shows the photographs of the flexible PCB coils.

## 4 Ex Vivo Validation

**FIGURE 5:**
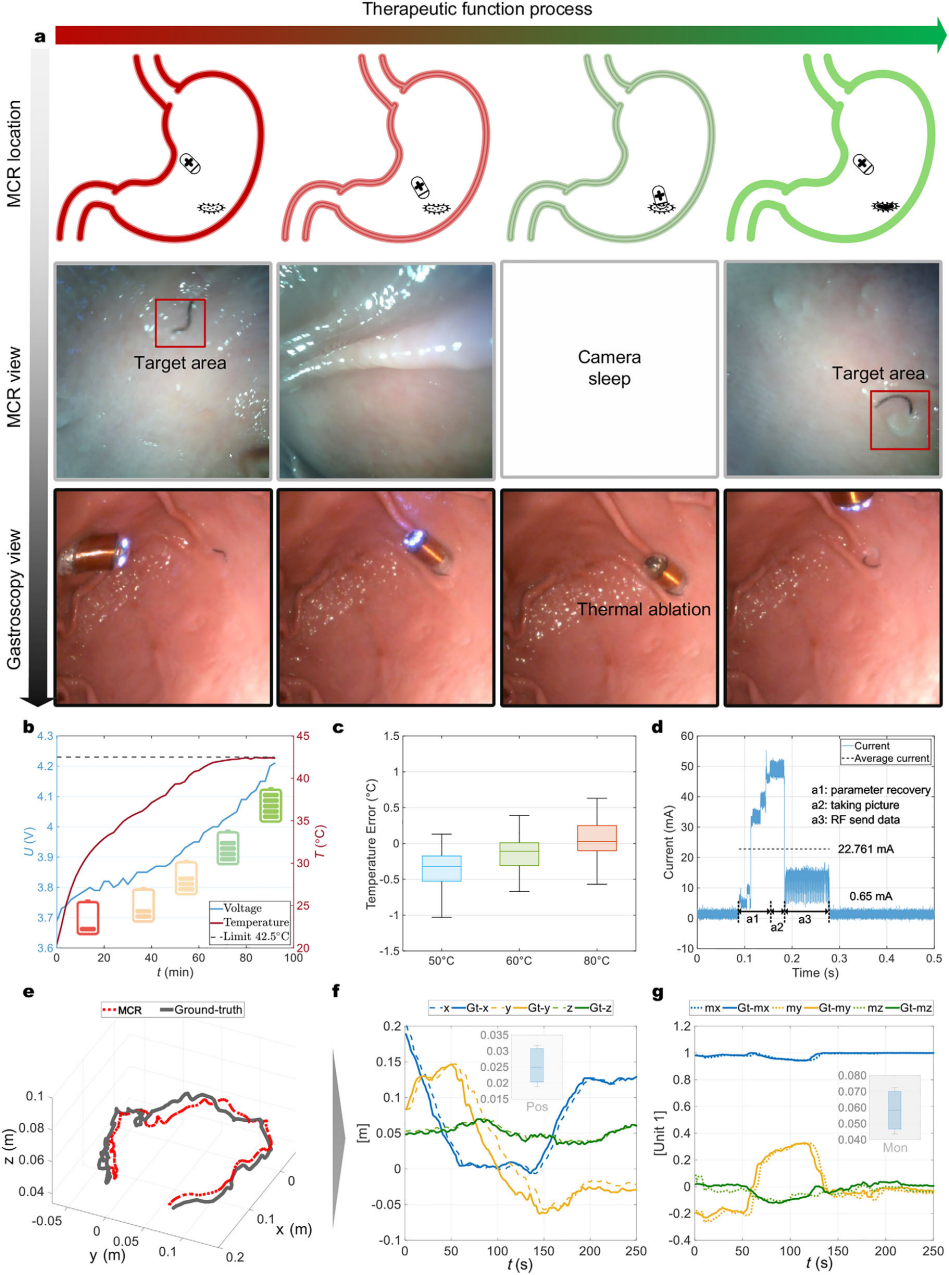
(a) Ex vivo experiment: identifying the target lesion, navigating to the site, performing localized thermal ablation, and departing for post‐treatment evaluation. (b) Variations in MCR temperature and battery voltage during wireless charging. The charging power is reduced as the MCR temperature approaches the predefined engineering control limit of 42.5 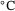. (c) Distribution of temperature control error during thermal ablation. d, Measured current consumption of the MCR operating at 2 FPS with 512×512 resolution. (e) Measurement results of the MCR localization test. (f) Localization error: discrepancy between MCR coordinates (dashed lines) and ground‐truth (solid lines), and error distribution. (g) Orientation error: discrepancy between MCR magnetic moment components (dashed lines) and ground‐truth (solid lines), and error distribution.

We conducted an ex vivo gastric‐wall tissue experiment to evaluate the MCR's integrated functionalities, including localization, wireless charging, image acquisition, and thermal ablation. Fresh porcine stomach tissue was obtained from a food supplier, rinsed thoroughly to remove residual contents, and kept moist at 4 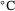 to preserve structural integrity. A small suture marker was placed on the internal stomach wall to define the target site. An air pump was used to maintain gastric inflation throughout the experiment. To enable continuous visual monitoring, a gastroscope was inserted through the cardia into the stomach lumen. The stomach was positioned inside a plastic container mounted on the magnetic actuation system, after which wireless power transfer and communication were calibrated and aligned. Using the MCR's onboard camera, visual navigation was performed, and the capsule's position was tracked using the magnetic localization system. MCR motion was externally guided via a robot‐manipulated permanent magnet. Real‐time images transmitted from the MCR allowed operators to monitor the procedure, while the gastroscope provided an independent view (Figure 5a). The capsule navigated toward the target using combined visual cues and magnetic pose estimation. Magnetic pose estimation provided coarse position and orientation information, while real‐time images from the capsule camera were used to guide the final approach. An additional gastroscope, which could be replaced by an additional capsule‐mounted camera in future implementations, provided independent visual confirmation that the capsule had reached the intended target region. Once positioned, the thermal ablation module was activated under predefined power settings. The MCR was held at the target for the planned duration (e.g., 120 s), during which temperature, power, and timing data were recorded. After thermal ablation, the MCR captured images of the treated region to document the outcome. During heating, the image‐transmission module operated in sleep mode or at a reduced frame rate to allocate maximum available power to the heating element.

Prior to performing thermal ablation, the MCR determines the energy availability and ensures sufficient energy to complete the process. Thermal ablation is enabled only when the state of charge (SOC) exceeds 60 %; If SOC falls below this threshold, the MCR automatically initiates wireless charging to recharge the battery. The MCR heating element draws 150 mA from the battery during thermal ablation, for 5 min. Given a fully charged battery of approximately 82 mAh (100 % SOC), a single thermal ablation consumes about 15.2 % SOC. At 60 % SOC of battery, the expected remaining SOC after ablation should be above 40 %, which is experimentally confirmed sufficient enough to support subsequent data acquisition, communications, and control operations under nominal conditions. Figure 5b presents the variations in battery voltage and MCR temperature during wireless charging. The charging time for the battery is approximately 90 min. The charging power reduces dynamically as the MCR body temperature approaches 42.5 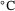 to ensure GI tract tissue safety. According to the Japan Society of Medical Electronics and Biological Engineering (JSMEBE), maintaining temperatures below this threshold minimizes the risk of thermal injury to surrounding tissue [41]. Figure 5c shows the performance of thermal ablation temperature control. The temperature deviation from setpoints of 50 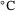, 60 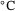, and 80 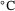 are shown. Figure 5d depicts the instantaneous current consumption of the MCR operating at 2 FPS with 512×512 resolution. Power consumption associated with various operations such as parameter recovery, taking a photo, and RF communication are clearly delineated. To assess localization accuracy, we implemented a magnetic localization algorithm within the MCR. Ground‐truth positions were simultaneously tracked using an optical tracker (Polaris Vega XT, RMS accuracy 0.15 mm at 250 Hz) via an external marker affixed to the MCR. Figure 5e compares the estimated magnetic trajectory with the ground‐truth trajectory. Quantitative analysis reveals a root‐mean‐square (RMS) positional error of 0.02495 m and a magnetic moment orientation error of 0.0585 (dimensionless), as shown in Figure 5f,g.

## 5 Assessment of Tissue and Cell Viability Following Thermal Ablation

**FIGURE 6:**
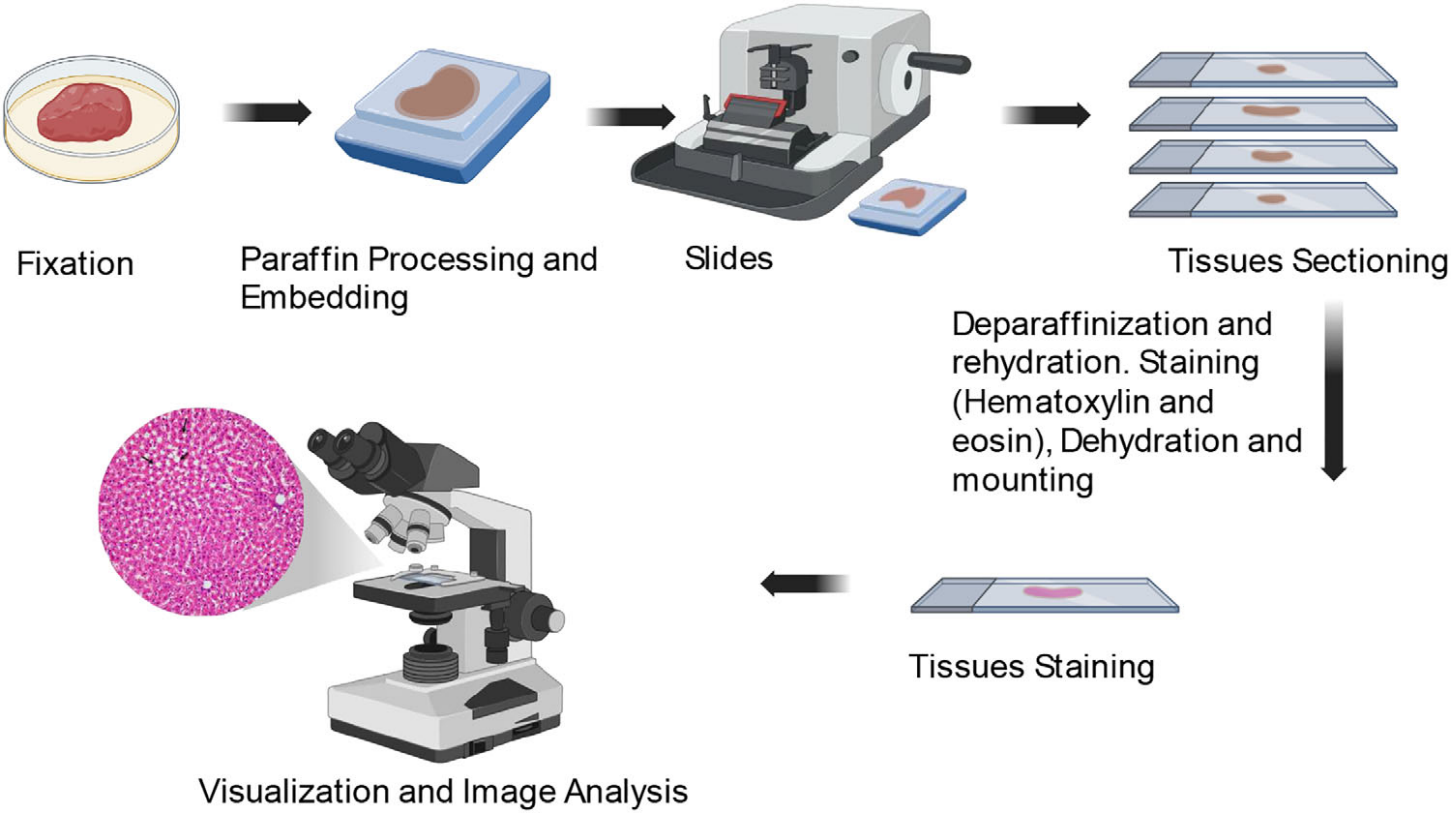
Workflow for histological analysis of tissue following thermal ablation. After treatment, tissue samples were fixed to preserve morphology, processed and embedded in paraffin, and sectioned horizontally into thin slices using a microtome. Sections were then deparaffinized, rehydrated, and stained with hematoxylin and eosin for structural assessment. Finally, stained slides were examined under a microscope to evaluate thermal‐induced tissue damage and compare treated and untreated regions.

**FIGURE 7:**
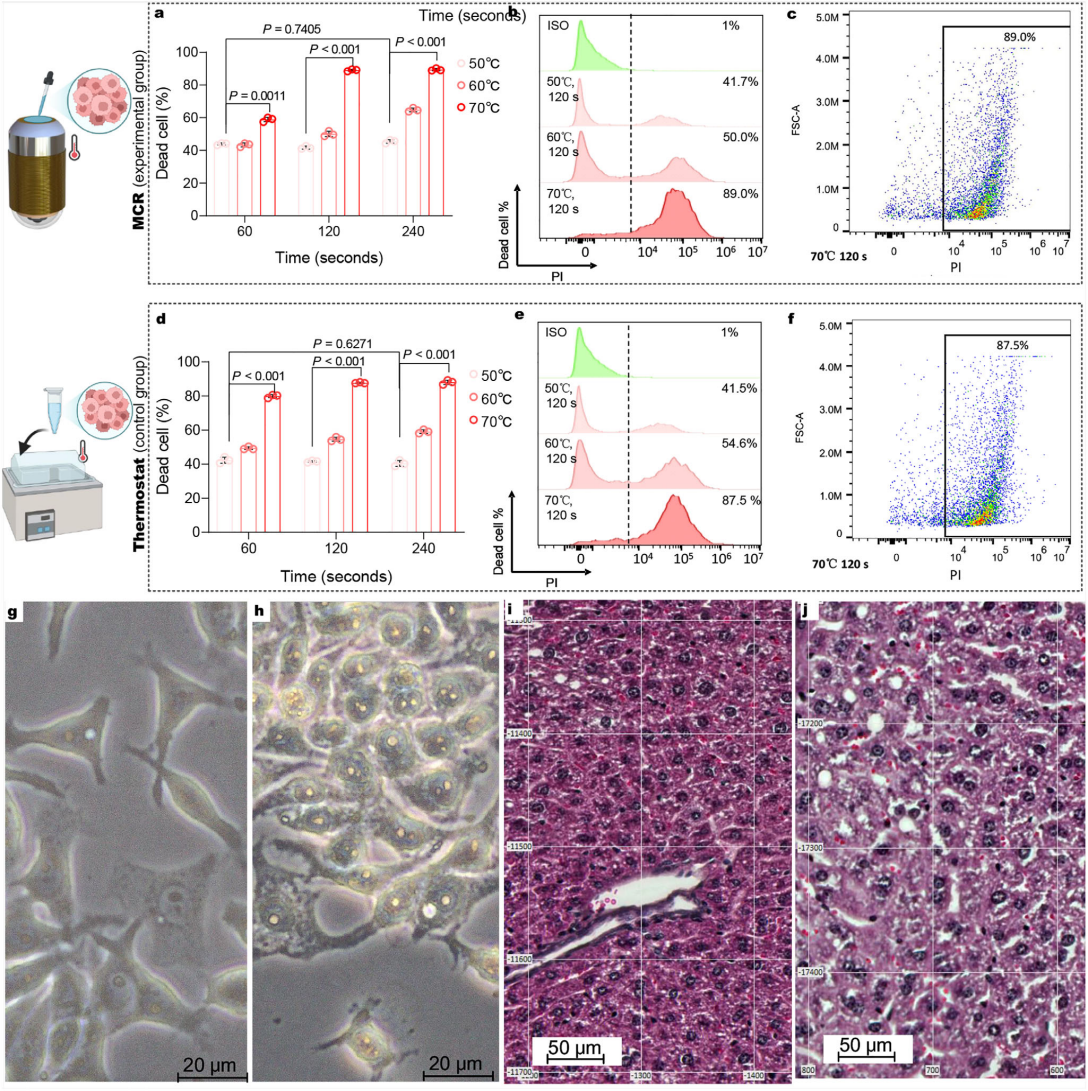
Assessment of HCT‐116 human colorectal carcinoma cell viability following MCR thermal ablation and thermostatic water bath treatment. (a, d) Quantitative analysis of dead cell percentage measured by flow cytometry, comparing cell necrosis after thermal ablation using the MCR and a laboratory thermostatic water bath at 50 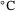, 60 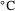, and 70 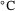 for 60, 120, and 240 s. Statistical analysis was performed using a three‐way factorial ANOVA with modality, temperature, and exposure duration as fixed factors, including all two‐way and three‐way interactions. Complete ANOVA results and Holm‐adjusted simple‐effects comparisons are provided in Tables S1 and S2, respectively. (b, e) Representative histogram plots of PI‐stained cells after thermal ablation at 70 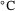 for 120 s using the MCR and thermostatic water bath, showing the proportion of dead cells (PI‐positive population). (c, f) Representative flow cytometry scatter plots (FSC‐A vs. PI) demonstrating high levels of necrotic cells following treatment (89.0% for MCR and 87.5% for thermostatic water bath at 70 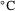 for 120 s). (g) Morphology of normal HCT‐116 human colorectal carcinoma cells. h, Morphology of HCT‐116 cells after MCR thermal ablation. (i, j) Histological staining of rat liver tissue: i, untreated control showing intact cellular morphology; (j) tissue after thermal ablation at 70 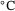 for 120 s, exhibiting pronounced cellular damage and necrosis.

We conducted a series of in vitro experiments to evaluate the effectiveness of the MCR's thermal ablation module in inducing cell mortality. In the first experiment, fresh rat liver tissue was used. Three liver samples were prepared, and the MCR was placed on the tissue surface to elevate the local temperature to 70 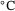 for 120 s. Following treatment, each sample was paraffin‐embedded, horizontally sectioned into 4 μm slices, and stained with hematoxylin and eosin. The stained sections were examined microscopically to compare treated and untreated regions and examine thermally induced tissue damage (Figure 6). Figure 7i. presents the untreated control tissue, while Figure 7j shows the thermally ablated sample. Clear morphological alterations are evident in the treated region, including cellular swelling, cytoplasmic loosening and hypochromasia, and the presence of fine intracellular vacuoles, likely reflecting edema or glycogen accumulation. These features collectively indicate that tissue underwent thermal injury following rapid temperature elevation.

To quantitatively assess the MCR's thermal ablation performance, a second experiment was conducted using HCT‐116 human colorectal carcinoma cells, an adherent cell line derived from colorectal cancer. Two groups of experiments were performed, comprising a total of 54 samples. In Group A, droplets of cell suspension were applied directly onto the MCR's heating pad and exposed to controlled temperatures of 50 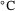, 60 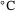, and 70 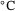. For each temperature, cells were heated for 60, 120, and 240 s, with three independent biological replicates per condition. Each biological replicate represented an independently prepared and treated cell sample. Technical measurements, where applicable, were averaged within each biological replicate and were not treated as independent observations. This setup allowed direct evaluation of the thermal ablation module's ability to induce cell mortality under defined thermal conditions. To provide a well‐controlled and homogeneous thermal reference, Group B samples were heated using a laboratory thermostatic water bath. Test tubes were first equilibrated to the target temperature, after which droplets of cell suspension were introduced and maintained at the corresponding temperature for the same exposure durations, ensuring uniform thermal distribution and minimized confounding factors. The water‐bath results served as a reference standard, validating the MCR's thermal ablation module performance and confirming that observed cell mortality was attributable primarily to thermal exposure, rather than mechanical, chemical, or handling‐related effects. After the experiments, cell viability was quantified using flow cytometry with live/dead fluorescent staining to distinguish intact from thermally damaged cells. The results are presented as percentage dead cells (Figure 7a,d), accompanied by representative histogram plots (Figure 7b,e) and flow cytometry scatter plots (Figure 7c,f). A three‐way ANOVA identified a significant modality–temperature–duration interaction ($F_{4,36}=51.10$, $P=2.34\times 10^{-14}$), indicating that the difference between the MCR and water‐bath treatments depended on temperature and exposure duration (Tables S1 and S2). Nonsignificant comparisons under selected matched conditions were not interpreted as evidence of equivalence. The data clearly show that temperature and exposure duration strongly influence ablation effectiveness. Lower temperatures (50 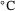
–60 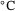) and short heating durations resulted in minimal cell mortality, indicating insufficient thermal dose. In contrast, cell death increased sharply when the temperature reached 70 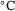 and the duration was extended from 60 to 120 s. Notably, the MCR achieved 89 % cell mortality after 120 s at 70 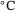, with only marginal additional benefit when the duration was further increased to 240 s. Using an inverted phase‐contrast microscope, morphological differences between untreated control cells and thermally ablated cells were also observed in Figure 7g,h, further confirming thermally induced cellular damage.

These findings highlight the high energy demand associated with effective thermal ablation and underscore the importance of providing uninterrupted power to the MCR via wireless power transfer. Overall, the experimental results provide preliminary support for the feasibility of achieving controlled thermal ablation using the MCR approach, while also indicating that further investigation is required to assess its practical performance and safety under in vivo conditions.

## 6 Discussion

Commercial capsule endoscopes are clinically validated devices designed primarily for passive imaging. They are optimized for low‐power consumption and extended operating time, and therefore, typically operate at modest frame rates (approximately 2–6 fps) to conserve battery energy. In contrast, the present work demonstrates a proof‐of‐concept multifunctional capsule robot capable of magnetic navigation and controlled thermal ablation under ex vivo and in vitro conditions. The incorporation of wireless power transfer addresses the energy constraints of the onboard batteries and enables exploration of higher‐power functionalities, including high‐frame‐rate imaging and active therapeutic modules.

**FIGURE 8:**
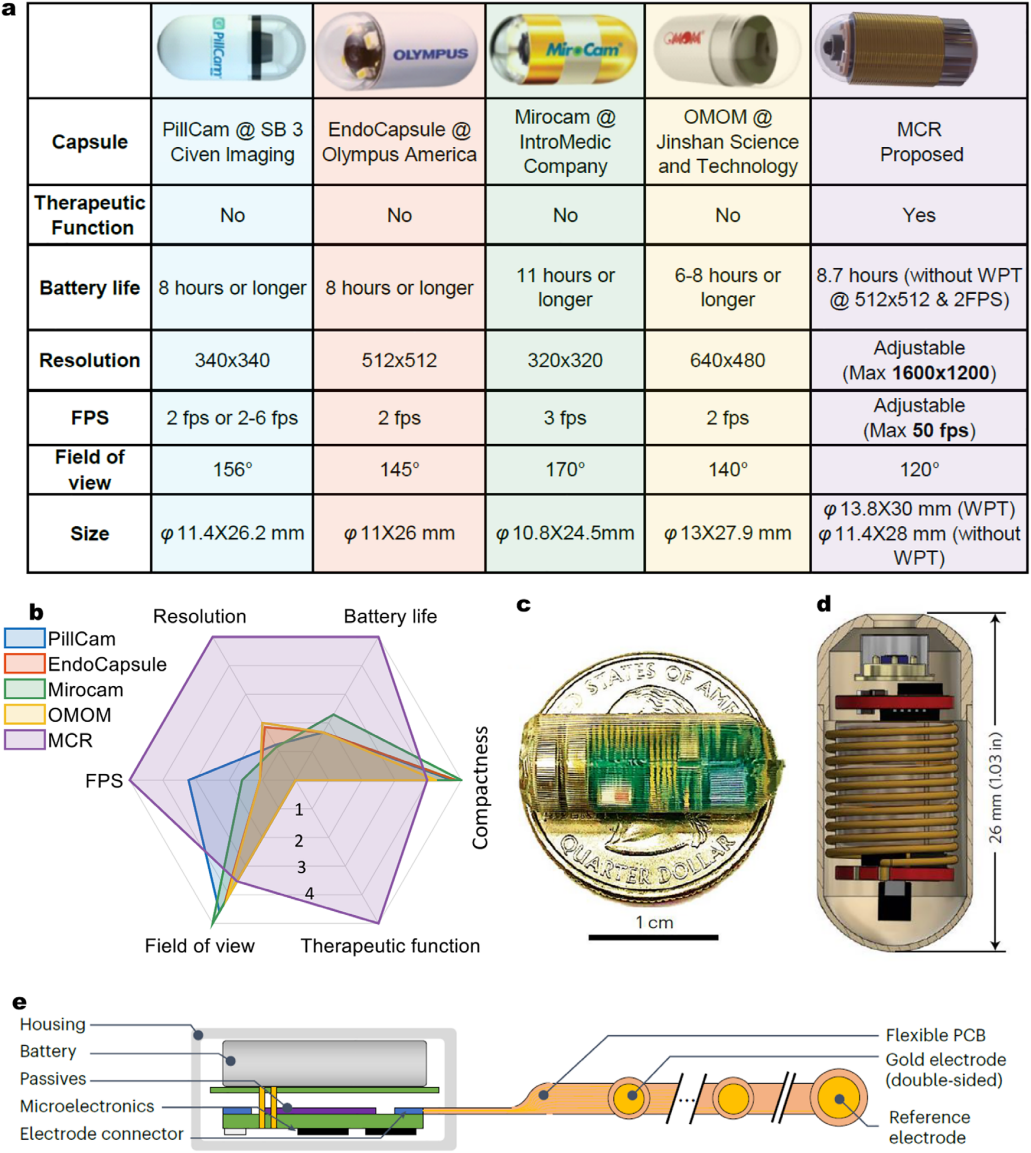
Contextual comparison of selected characteristics of commercial capsule endoscopes and the proposed MCR. (a) Reported system parameters. (b) Radar chart showing normalized attribute profiles on a 0–5 scale. Battery life, FPS, and field of view were normalized as $S_{i}=5x_{i}/x_{\max}$, where $x_{i}$ is the value listed in this figure a and $x_{\max}$ is the largest value among the compared devices. Battery life for the commercial devices was based on reported specifications, whereas the MCR value was based on its measured onboard operating time without WPT. Resolution was normalized as $S_{i}=5\sqrt{N_{i}/N_{\max}}$, where $N_{i}$ is the total pixel count. Compactness was calculated as $S_{i}=5{\left(V_{\min}/V_{i}\right)}^{1/3}$, where $V_{i}$ is the capsule volume. Therapeutic functionality was coded as 5 when present and 0 when absent. (c) Location‐aware ingestible microdevices for wireless monitoring of gastrointestinal dynamics (Nature Electronics, 2023) [46]. (d) A human pilot trial of ingestible electronic capsules capable of sensing different gases (Nature Electronics, 2018) [47]. (e) An ingestible device for gastric electrophysiology (Nature Electronics, 2024) [48].

Figure 8a summarizes key system‐level parameters of commercial capsule endoscopes and the proposed MCR. This comparison is intended solely to contextualize the functional scope of the prototype, rather than to imply performance advantages over established clinical devices. Because the source specifications were obtained or reported under different operating conditions, the comparison is intended only for contextual visualization; neither the individual normalized values nor the radar‐chart area represents an overall performance measure. Figure 8b presents a radar chart illustrating differences in design characteristics such as compactness, battery life, and imaging capability. As a research prototype, the system uses off‐the‐shelf components and manual assembly to enable rapid iteration and feasibility testing. A clinical‐grade device, however, will require substantial re‐engineering, including optimization of the wireless power transfer architecture (e.g., coil and ferrite design), improved optical performance, higher‐precision localization and pose control, further miniaturization, and fully integrated circuitry suitable for automated, medical‐grade manufacturing. The present design also does not evaluate in vivo navigation, long‐term biocompatibility, thermal‐safety margins in living tissue, or integration into clinical workflows.

The proposed MCR is positioned as a research platform exploring how additional functionalities—such as magnetic actuation, wireless power transfer, and targeted thermal ablation—may be incorporated into future capsule systems. Recent developments in advanced capsule technologies further highlight this direction. Figure 8c shows the location‐aware capsule by Sharma et al., designed for motility monitoring in patients with fecal incontinence. Figure 8d illustrates the gas‐sensing capsule developed by Kalantar–Zadeh et al., capable of detecting oxygen, hydrogen, and carbon dioxide within the gastrointestinal tract. Figure 8e presents the multi‐contact voltage‐sensing capsule by You et al., which records electrical activity inside the digestive system. These examples demonstrate the growing interest in expanding capsule functionality beyond imaging and underscore the need for continued innovation.

Within this context, the proposed MCR contributes two exploratory capabilities: (1) pose control via manipulation of an external permanent magnet, enabling improved localization and orientation for imaging, and (2) localized thermal ablation, which may allow targeted intervention when abnormal tissue is identified. These functions are relevant to the long‐term vision of minimally invasive GI therapeutic systems, although substantial further development and validation are required before clinical translation.

Despite the new functionalities demonstrated by the proposed MCR, several limitations remain. The current localization accuracy decreases substantially when the capsule moves beyond the effective sensing range of the Hall‐effect array, as reported in our previous work [42]. This limitation may affect target‐placement accuracy when the capsule is positioned far from the sensor plane. Future development will focus on integrating visual navigation with magnetic sensing. Capsule image sequences may provide complementary information, including lumen direction, tissue texture, and frame‐to‐frame motion. Fusion of these visual cues with Hall‐sensor measurements may improve the robustness and accuracy of capsule pose estimation under complex gastrointestinal conditions.

In addition, further miniaturization of the capsule is required to enhance swallowability and ensure natural excretion without discomfort. However, reducing the device size introduces several predictable engineering challenges. First, wireless power transfer efficiency decreases as coil dimensions shrink and magnetic coupling weakens. Second, thermal density increases because miniaturised electronics have less surface area for heat dissipation, raising the risk of local overheating. Third, maintaining optical performance becomes more difficult as smaller apertures and reduced sensor area can degrade image quality and signal‐to‐noise ratio. Finally, biocompatible encapsulation adds unavoidable thickness to the outer shell, reducing internal usable volume and further constraining space for coils, optics, electronics, and any energy‐storage components.

It should be noted that the present validation is restricted to controlled ex vivo and in vitro settings, which do not fully capture the dynamic and heterogeneous in vivo gastrointestinal environment. Factors such as variable tissue compliance, peristaltic motion, and fluidic disturbances can affect the precision and stability of localization performance, while the presence of mucus and gastric fluids may influence thermal energy delivery. Establishing well‐defined thermal safety protocols will therefore be essential to prevent unintended injury to surrounding tissues in future in vivo studies.

Looking ahead, the proposed MCR also provides an open research platform for integrating emerging advances in biomedical imaging and AI‐assisted diagnostics. Autonomous navigation strategies supported by real‐time sensing and control algorithms may enhance the capsule's ability to reach and maintain position at target sites under complex physiological conditions [43]. In addition, recent image‐processing advances in capsule endoscopy, such as narrow‐band imaging algorithms based on decorrelated color space, may improve the detection and classification of early gastrointestinal lesions [44]. Advanced hyperspectral imaging combined with AI‐assisted analysis also shows potential for enhancing the classification of selected gastrointestinal diseases [45]. In future system upgrades, deep‐learning frameworks may be further combined with active magnetic manipulation to identify suspicious or insufficiently visualized regions and guide repeated observation. Together, these directions outline a pathway toward more capable and intelligent capsule robotic systems, while emphasizing that substantial engineering and biological validation remain necessary before clinical translation.

## 7 Conclusion

In this work, we present a multifunctional capsule robotic system that integrates diagnosis and targeted therapy within a single platform. Its capabilities are validated through ex vivo gastrointestinal tissue experiments and in vitro cellular assays. Comprehensive ex vivo evaluations demonstrate that the MCR can acquire diagnostic images, maintain stable wireless communication and localization, and execute controlled motion. Building on these core functions, the MCR performs site‐specific thermal ablation of the gastric wall under ex vivo conditions using combined visual guidance and a magnetic control system. Complementary in vitro experiments showed that the thermal ablation module can induce substantial cellular destruction, achieving up to 89% cell mortality under controlled heating conditions. To address the substantial energy demands of thermal ablation, the system incorporates wireless power transfer, enabling sustained operation without dependence on limited onboard battery capacity. While the current study is restricted to ex vivo and in vitro validation, it provides a proof‐of‐concept demonstration for the future development of a multifunctional capsule‐based therapeutic system and supports subsequent investigation toward in vivo studies and eventual clinical translation.

## 8 Method

### 8.1 Prototyping

The internal configuration of the proposed MCR is illustrated in Figure 2a. The device comprises a camera lens, electronic circuitry, rechargeable batteries, a permanent magnet, a heating element, and a wireless power receiving coil. All the electronic components are integrated on a tailor‐made double‐sided flexible printed circuit board (FPCB), fabricated as a standard 2‐layer, 0.2 mm thick board with 1 oz/${\mathrm{ft}}^{2}$ copper on a 25 μm polyimide substrate. The FPCB is divided into five sections: wireless power module, microcontroller unit (MCU) module with radio frequency (RF) communication, an inertial measurement unit (IMU) module, an image sensor module, and an LED illumination module. Each section is designed to fit within a 9.9 mm diameter, allowing the board to be folded into a compact cylindrical form. After component assembly and folding, the FPCB forms a cylinder measuring 9.9 mm in diameter and 10.4 mm in length.

A 5.5 mm diameter, 3.2 mm long camera lens is mounted on top of the image sensor using a lens holder. On the opposite end, two batteries (Lir1054 and Lir1040) are connected in parallel, forming a single power module approximately 10 mm in length and 10 mm in diameter. The assembly is housed within a ferrite core with an outer diameter of 13 mm, an inner diameter of 11 mm, and a length of 16 mm. The ferrite is tightly wound with 130 turns of 0.19 mm diameter enameled copper wire. The camera side of the MCR is sealed with a hemispherical quartz glass shell, while the battery side is attached to a 13 mm diameter, 5 mm long strong neodymium magnet to enable magnetic positioning, translational and rotational motion control of the MCR.

A 3mm thick organic polymer layer with low thermal conductivity of 0.1–0.5 W/(m·K) is inserted between the heating element and the batteries as the thermal insulation barrier. The heating element consists of a custom‐made 8 mm diameter heating plate with an onboard negative temperature coefficient (NTC) temperature sensor. The entire assembly is finally encapsulated with a layer of Biaxially Oriented Polypropylene (BOPP) film to ensure durability and structural stability. After full assembly, the MCR has an outer diameter of 13.8 mm and a length of 30 mm.

### 8.2 MCU and RF Communication

The microcontroller (CH32V208CBU6), equipped with RF communication, supports a wireless transmission rate of 2 Mbps and operates at a maximum clock frequency of 144 MHz. It includes 128 KB of Flash memory and 64 KB of RAM. To enable image transmission, the MCU divides each captured image into multiple smaller packets. Each packet carries a maximum payload of 251 bytes and contains the total number of packets, the packet index, and the corresponding image data. All packets follow a protocol‐defined frame structure that includes the device address and a cyclic redundancy check (CRC) for detecting accidental data corruption. The protocol also incorporates acknowledgment and retransmission mechanisms, improving data reliability and enhancing robustness against external electromagnetic disturbances. Upon receiving each packet, the RF receiver sends an acknowledgment to confirm successful reception, prompting transmission of the next packet. The communication protocol ensures reliable, orderly, and efficient data transfer. Experimental measurements show that the maximum achievable data rate is approximately 1.8 Mbps.

A dedicated RF receiver (Figure S2c.) was developed to support bidirectional communication. It receives image and sensor data transmitted from the MCR and also relays control commands back to the device. The received data can be forwarded to a laptop via a serial interface or transmitted to a portable device. The portable device (Figure S2d.), developed as part of the system, incorporates an STM32H743VIT6 processor and a 2.8‐inch display for real‐time visualization of images and sensor data. It also includes a TransFlash (TF) card connected through the secure digital input output (SDIO) interface for image storage.

The system supports dual‐mode operation: after receiving image data in RF mode, the receiver can be reconfigured to operate in Bluetooth low energy (BLE) mode, enabling seamless transmission of images directly to a smartphone. This dual‐mode capability enhances flexibility in data transfer and broadens the potential applications of the portable device.

### 8.3 Image Sensor

The image sensor (OV2640) supports a maximum resolution of 1600 × 1200 pixels at 15 FPS in UXGA mode and up to 60 FPS in CIF mode (352 × 288). It provides direct JPEG output and has a compact footprint of 5.7×6.3×0.9 ${\mathrm{mm}}^{3}$. The OV2640 is controlled by the MCU via an I2C interface, which manages resolution settings and other operational parameters. Image data are transferred from the sensor to the MCU through eight parallel data lines and a dedicated pixel‐clock signal. To improve power efficiency, the OV2640 includes a control pin that enables rapid transition into standby mode during idle periods, thereby reducing energy consumption. Under diagnostic operating mode, the system supports continuous image acquisition at 2 FPS with a resolution of 512×512 pixels for up to 8 h without recharging [49].

The lens is surrounded by six LEDs, and a light sensor is incorporated to provide optical activation for the MCR. In standby mode, the device remains inactive until the light sensor detects illumination from an external source, triggering system activation. This optical triggering mechanism eliminates the need for mechanical switches, improving hermetic sealing and reducing structural complexity. The light sensor's compact 0603 package further supports space‐constrained integration. The six LEDs are driven by a MOSFET (PMXB56ENZ), which powers the illumination only during image capture to conserve energy. This MOSFET features a minimal footprint of 1.1×1.0×0.37 ${\mathrm{mm}}^{3}$, contributing to the overall compactness and efficiency of the imaging module.

### 8.4 Heating Element

The thermal ablation module integrates a custom‐designed heating element, a MOSFET, and a negative temperature coefficient (NTC) thermistor for temperature sensing. The heating element, with a resistance of 26 Ω, is embedded within an 8 mm diameter heating pad to ensure localized thermal delivery. The customized heating pad (JLC Heating Film, Shenzhen JLC Technology Group Co., Ltd., Shenzhen, China) comprised a 0.03‐mm‐thick FeCrAl resistive track, with a width of 0.21 mm and a total length of approximately 106.01 mm, sandwiched between two 0.05‐mm‐thick PI insulating films, resulting in a nominal total thickness of 0.13 mm. The FeCrAl pattern was formed by chemical etching and subsequently laminated with the polyimide films under elevated temperature and pressure; the heater was rated at 4.0 V and 0.60 W, and the NTC thermistor was integrated by the same manufacturer. A PMXB56ENZ MOSFET regulates the power supplied to the heating element. Temperature control is achieved by adjusting the MOSFET duty cycle through pulse‐width modulation (PWM) generated by the MCU, enabling precise regulation of the heating temperature.

The NTC thermistor, with a nominal resistance of 100 kΩ (1% accuracy) and a B‐value of 3950 K (25 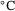/50 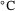), is packaged in a compact 0402 form factor, providing accurate and responsive thermal feedback. Its temperature‐dependent resistance is converted into a voltage signal and fed back to the MCU for closed‐loop temperature regulation. For improved energy efficiency, the NTC sensor is also controlled by a PMXB56ENZ MOSFET, which remains deactivated during standby mode to minimize power consumption. This configuration effectively reduces standby energy usage while maintaining reliable thermal ablation control during active operation.

### 8.5 Power Conversion and Battery

The use of Low‐dropout (LDO) regulators, although advantageous in terms of compact size and low component count, is not recommended in this application due to their relatively higher energy loss. The MCU and RF communication modules require a 2.5 V supply, derived from the 3.3 V battery using a high efficiency DC‐DC converter. The image sensor requires two voltage levels, 1.2 V and 2.5 V, both provided by dedicated DC‐DC converter modules to ensure optimal power efficiency and stability. These converters employ the BD70522GUL chip, which features an ultra‐low quiescent current of 180 nA and achieves up to 90% efficiency at a 10 μA output current. This chip is packaged in a compact 1.76×1.5×0.57 ${\mathrm{mm}}^{3}$ footprint.

Two Li‐ion batteries, Lir1054 and Lir1040 Li‐Ion batteries were selected for their compact size, rechargeability, and suitability for capsule integration. The Lir1054 provides 48 mAh (10 mm diameter, 5.4 mm height), while the Lir1040 provides 34 mAh (10 mm diameter, 4.0 mm height). When connected in parallel, they offer a combined capacity of approximately 82 mAh. This configuration enables the MCR to operate continuously for up to 8.7 hours at 512×512 resolution and 2 FPS without wireless charging. Battery status is monitored by the MCU using a voltage divider composed of two 100 kΩ resistors connected in series. To minimize power consumption, the voltage divider remains open‐circuit and is activated only via a MOSFET switch under MCU control.

### 8.6 Power Consumption and Wireless Charging

The MCR operates in two modes: standby mode and work mode. In standby mode, the device consumes approximately 2 μA and remains idle until activation. Once activated, it switches to work mode, capturing images at 1 FPS with a resolution of 512×512. After taking a capture, the image is transmitted via RF, and the MCR immediately enters a sleep state to conserve energy. In standby mode, all DC‐DC power supplies are disabled except the MCU's converter, allowing the MCU to remain in an ultra‐low‐power state. Transitioning from standby to active mode is triggered by light detection. In active mode, the OV2640 camera module employs adaptive power management strategies based on the capture interval: for intervals shorter than 3 seconds, the camera enters a low‐power standby state via its dedicated pin, enabling rapid resumption of image capture; for intervals longer than 3 seconds, the camera's DC‐DC supply is disabled to conserve energy, requiring reinitialization upon wake‐up. The camera module consumes a peak power of 140 mW and a standby current of approximately 0.6 mA. Power consumption of measurements shows a peak current of 55 mA (at 3.8 V) and an average of 46 mA during continuous image capture (512×512 at 9.3 FPS) and RF transmission. At lower frame rates, the current decreases to 9.1 mA at 2 FPS and 4.9 mA at 1 FPS. Under intermittent capture, the average current drops significantly, measuring 2.05 mA for a 3‐second capture cycle and 0.65 mA for a 10‐second cycle at the same resolution. The MCR standby power consumption remains below 2 μA, ensuring extremely low‐energy operation.

During wireless charging, high‐frequency AC power at 218 kHz is magnetically coupled from the external transmitting coil to the MCR's receiving coil. The receiving coil incorporates a ferrite core made from TPG33B material. TPG33B offers high permeability ($\mu_{i}$=3500) and low loss, significantly improving power‐reception efficiency. At the operating frequency, the coil exhibits an inductance of 258 μH and a resistance of 15.772 Ω. After rectification and filtering, the AC power is converted to DC and used to supply power to the electronic modules via DC–DC converters, which then charge the batteries. The MCR draws energy directly from the charging coil, while excess energy is stored in the batteries to extend operational endurance and maintain functionality during charging interruptions.

### 8.7 Software

Two host‐computer software applications were developed in Visual Studio 2022: one dedicated to displaying and saving image data, and the other designed to record sensor information—such as acceleration and temperature—and to remotely modify MCR parameters including frame rate, resolution, and RF transmit power. Both the RF receiver and the MCR's MCU were programmed using MounRiver Studio. Data analysis was performed in MATLAB (R2022a). Illustrations were created using Adobe Illustrator (2022) and Microsoft Vision.

## Author Contributions

H.Z., C.K.L., Y.S., and Z.L. conceived and designed the study. H.Z. developed and assembled the capsule device, transmitting coils, and wireless charging system. Y.S. performed the localization experiments. H.Z. and Y.S. jointly conducted the ex vivo gastric tests and thermal ablation procedures. X.Z. and H.C. designed and conducted all cell‐based and tissue experiments. Y.L. contributed to the literature review and drafted the Introduction and Abstract. C.K.L. performed a comprehensive edit of the final manuscript. P.M. provided input on the Discussion and outlined directions for future work.

## Ethics Statement

All animal experiments in this study were approved by the Animal Experimentation Ethics Committee of the Chinese University of Hong Kong (Ref. No.22‐045‐NSF).

## Conflicts of Interest

The authors declare no conflicts of interests.

## Supporting information


**Supporting File**: advs77508‐sup‐0001‐SuppMat.pdf.
