# Supplementary material for: A Preclinical Multifunctional Capsule Robot with Unified Diagnosis and Targeted Thermal Therapy for Gastrointestinal Diseases
Source: Adv Sci (Weinh). 2026 Sep 6:e77508. Online ahead of print. doi: 10.1002/advs.77508 (PMC13547721; doi:10.1002/advs.77508)
Supplement: Supplementary file 1 [file ADVS-9999-e77508-s001.pdf]

Supplementary

A

A.1 Statistical analysis of the cellular experiments

The cell-death data were analyzed using a three-way factorial ANOVA, with treatment modality (MCR or thermostatic water bath), temperature (50, 60, or 70 °C), and exposure duration (60, 120, or 240 s) as fixed factors, including all two-way and three-way interactions. Each of the 18 experimental conditions included three independent biological replicates ($n = 3$ per condition; 54 observations in total). Each biological replicate represented an independently prepared and treated cell sample. Repeated measurements from the same sample were treated as technical replicates and averaged before statistical analysis. The complete ANOVA results are reported in Table S1.

Because the three-way interaction was significant, simple-effects comparisons between the MCR and water-bath treatments were performed within each matched temperature–duration condition using the pooled residual variance from the three-way ANOVA. The resulting P values were adjusted across the nine comparisons using the Holm method and are reported in Table S2.

Table S1 Three-way factorial ANOVA of dead-cell percentages following MCR and thermostatic water-bath treatments. The percentage of total variation was calculated as the sum of squares for each effect divided by the total sum of squares.

Source	SS	df	MS	F	Exact P	Total variation (%)
Modality	26.460	1	26.460	14.516	5.23×10^{-4}	0.151
Temperature	14508.041	2	7254.021	3979.652	5.79×10^{-43}	82.693
Exposure duration	1230.803	2	615.402	337.617	4.76×10^{-24}	7.015
Modality × temperature	113.154	2	56.577	31.039	1.46×10^{-8}	0.645
Modality × duration	368.068	2	184.034	100.963	1.73×10^{-15}	2.098
Temperature × duration	859.659	4	214.915	117.905	3.65×10^{-20}	4.900
Modality × temperature × duration	372.594	4	93.149	51.103	2.34×10^{-14}	2.124
Residual	65.620	36	1.823	–	–	0.374
Total	17544.400	53	–	–	–	100.000

Table S2 Simple-effects comparisons between the MCR and thermostatic water-bath treatments under matched temperature–duration conditions. Values are mean ± SD ($n = 3$ independent biological replicates per condition). Mean difference was calculated as MCR minus water bath. P values were adjusted across the nine comparisons using the Holm method.

Temperature (°C)	Duration (s)	MCR dead cells (%)	Water-bath dead cells (%)	Mean difference	Holm-adjusted P
50	60	43.93 ± 0.64	42.30 ± 1.75	1.63	0.44138
50	120	41.70 ± 0.89	41.53 ± 0.50	0.17	0.88067
50	240	45.43 ± 1.06	40.43 ± 1.72	5.00	3.07×10^{-4}
60	60	43.33 ± 1.19	49.37 ± 0.81	−6.03	2.81×10^{-5}
60	120	50.07 ± 1.65	54.63 ± 1.17	−4.57	7.94×10^{-4}
60	240	64.77 ± 1.12	59.13 ± 1.11	5.63	7.51×10^{-5}
70	60	58.87 ± 1.33	79.87 ± 0.93	−21.00	1.96×10^{-19}
70	120	89.03 ± 0.57	83.80 ± 3.24	5.23	1.95×10^{-4}
70	240	89.37 ± 0.72	88.03 ± 1.26	1.33	0.46868

A.2 Flow-cytometry gating and ex vivo histological assessment

A representative flow-cytometry gating strategy is shown in Fig. S1. The main cell population was first selected using FSC-H versus SSC-H to exclude debris, followed by singlet selection using FSC-A versus FSC-H to exclude doublets. PI-positive cells were then identified within the singlet population. The PI threshold was determined using unstained and heat-killed control samples, and the dead-cell percentage was calculated as the proportion of PI-positive events among gated singlets.

Three independent ex vivo experiments were performed using fresh liver specimens obtained from three rats. For each specimen, the tissue directly beneath the capsule heating pad was defined as the treated region, while an unheated region of the same specimen served as the control. Histological assessment was performed under blinded conditions. Procedural success was defined as maintaining contact between the heating pad and the tissue, reaching 70 °C, and completing the 120-s heating period without interruption. All three experiments met these criteria.

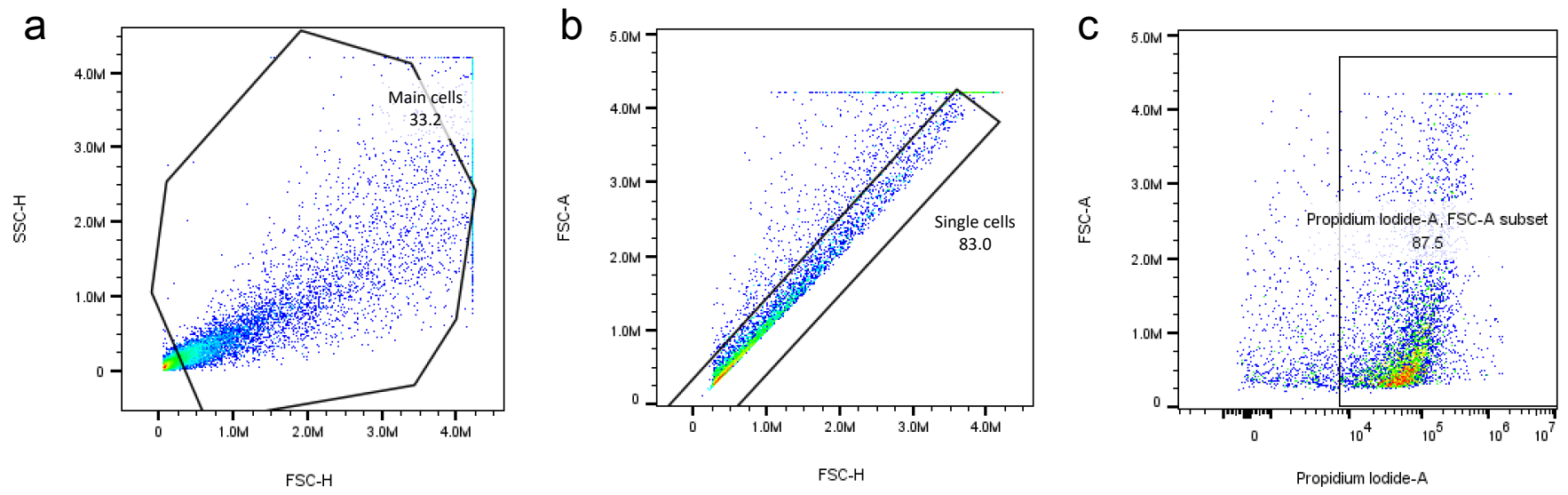

Fig. S1 Representative flow-cytometry gating strategy for HCT-116 cells. a, Selection of the main cell population using FSC-H versus SSC-H to exclude debris. b, Singlet selection using FSC-A versus FSC-H to exclude doublets. c, Identification of PI-positive cells within the singlet population. The PI threshold was determined using unstained and heat-killed control samples.

A.3 MCR details

A block diagram of the MCR's internal connections is presented in Fig.S2a. The application scenario of the MCR is illustrated in Fig.S2b. An RF receiver with serial communication is connected to a personal computer or a portable device (e.g., a mobile phone), as shown in Fig.S2c. The portable device displaying diagnostic images and sensor data, including location information is shown in Fig.S2d.

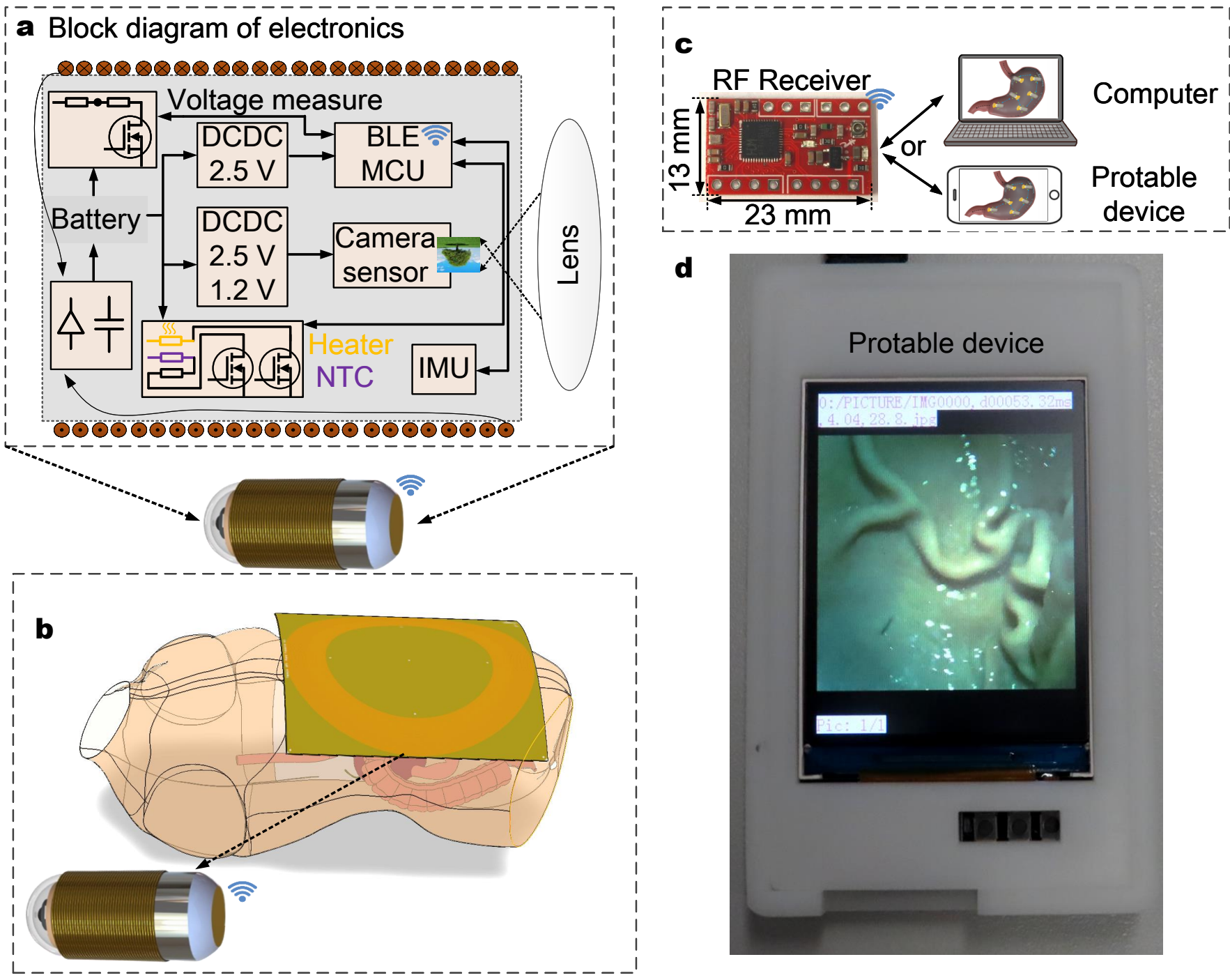

Fig. S2 Block diagram of the MCR's internal connections, the application scenario, and the external interface components. a, Internal system architecture of the MCR. b, Application scenario illustrating how the MCR operates within the diagnostic workflow. c, RF receiver connected to a personal computer or portable device via serial communication. d, Portable device displaying diagnostic images and sensor data, including location information.

Captured time-domain sensor data—including IMU readings, temperature, battery voltage, and received signal strength indicator (RSSI)—at a resolution of 512 × 512 and a frame rate of 2 FPS are shown in Fig. S3. The photo-taking current waveforms of the OV2640 under different control modes are presented in Fig. S4. The maximum frame rates achieved by the MCR at various resolutions are summarized in Table S3.

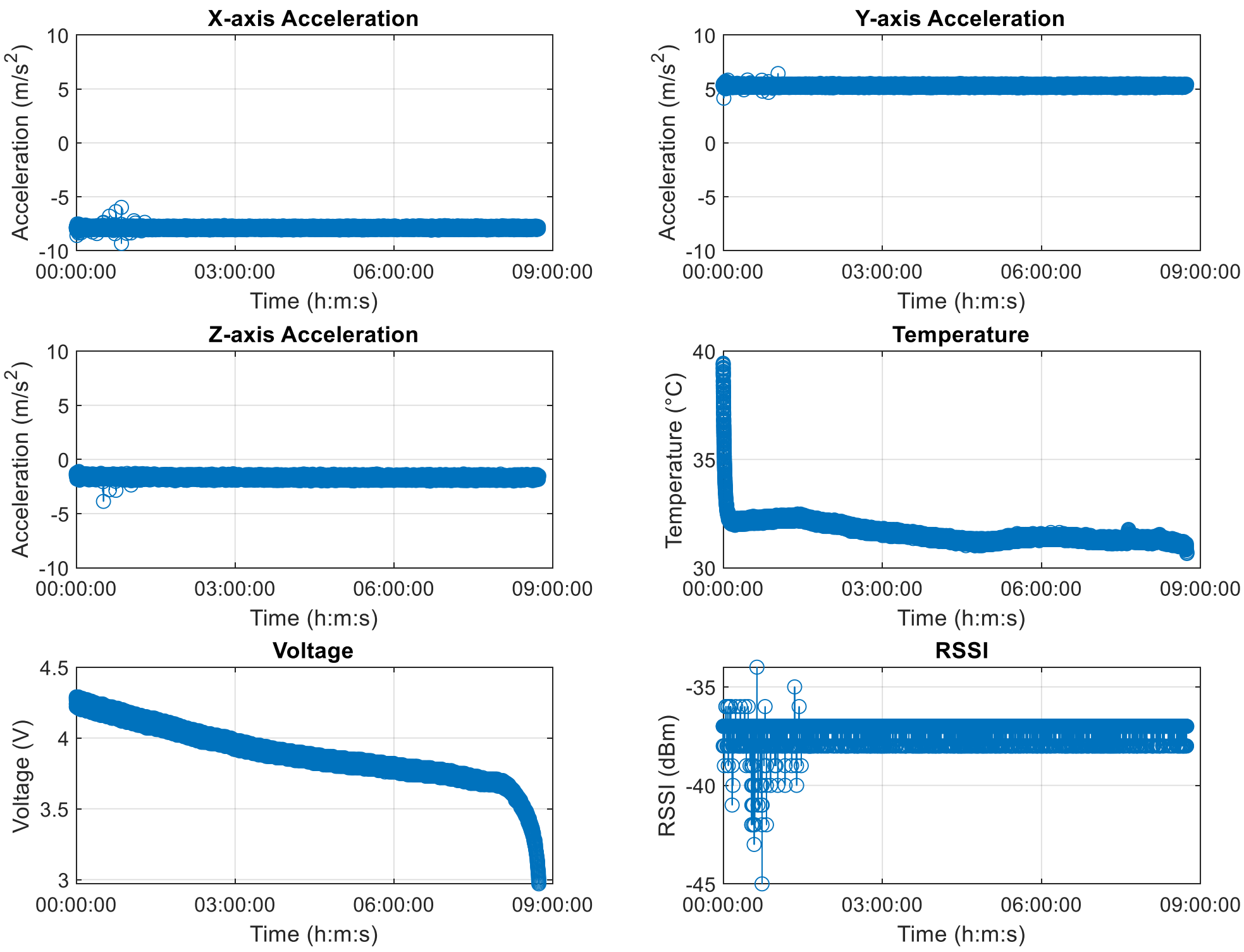

Fig. S3 Captured time-domain sensor data from the MCR, including IMU signals, temperature, battery voltage, and received signal strength indicator (RSSI), recorded at a resolution of 512×512 and a frame rate of 2 FPS.

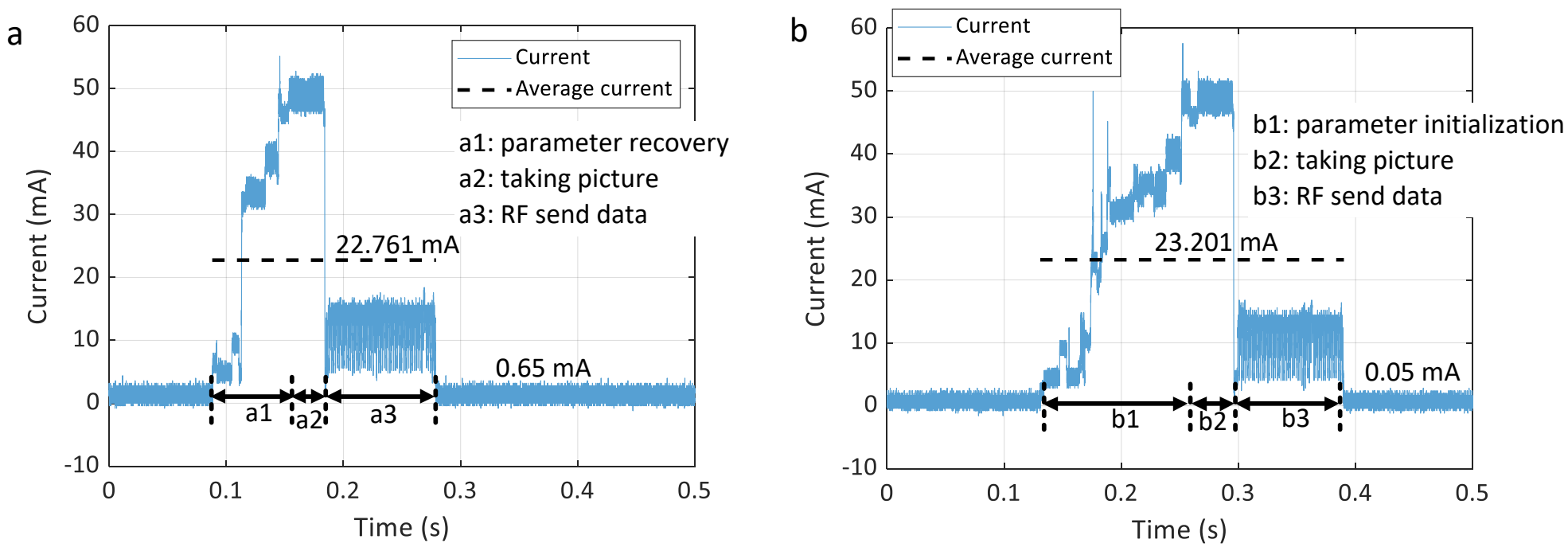

Fig. S4 The current consumption of the OV2640 image sensor under different power-management control modes. a, Transition from standby mode to image capture followed by data transmission. b, Transition from power-up to image capture followed by data transmission.

Table S3 MCR FPS at different image resolutions.

Format		Max FPS
CIF	160x120	49.5
SVGA	160x120	27.5
SVGA	320x240	27.5
SVGA	352x288	27.3
SVGA	512x512	9.3
SVGA	640x480	9.3
UXGA	800x600	7.3
UXGA	1024x768	5.0
UXGA	1024x1024	3.8
UXGA	1280x1024	3.8
UXGA	1600x1200	2.5-3

The relationship between the current consumption of the MCR and the picture-taking cycle under different imaging modes is shown in Fig. S5. The scope of targeted therapy is illustrated as shown in Fig. S6.

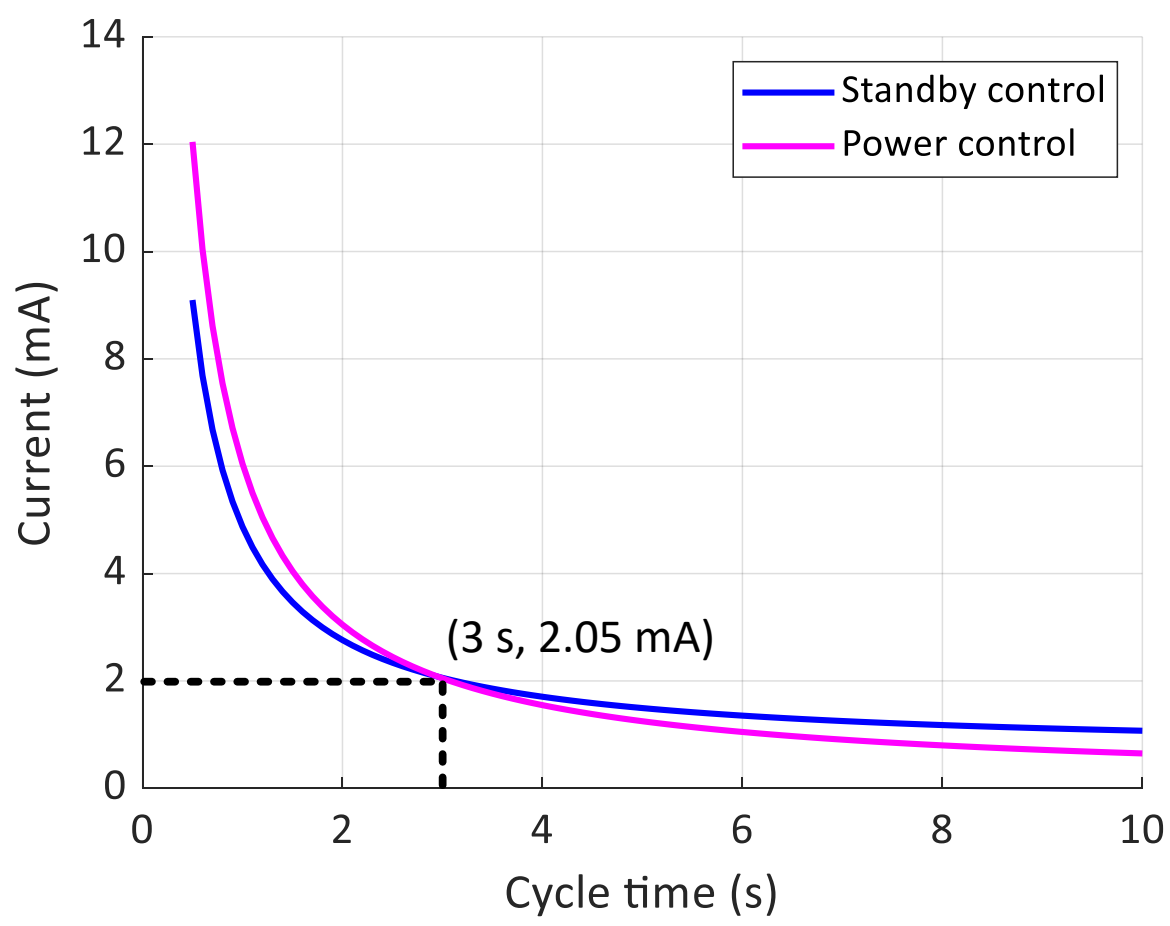

Fig. S5 Relationship between current and picture-taking cycle in different control modes.

A numerical analysis of thermal spread within surrounding tissue was performed using the Pennes bioheat equation[1]. The MCR heating element was modeled as an 8 mm-diameter circular heat source maintained at 70 °C and placed in contact with tissue initially at 37 °C. The model incorporates thermal conduction, perfusion-mediated heat dissipation, and metabolic heat generation. The resulting steady-state temperature distribution is shown in Fig. S7a, illustrating the spatial temperature gradient around the heat source. Fig. S7b presents the temperature decay along the central axis as a function of tissue depth.

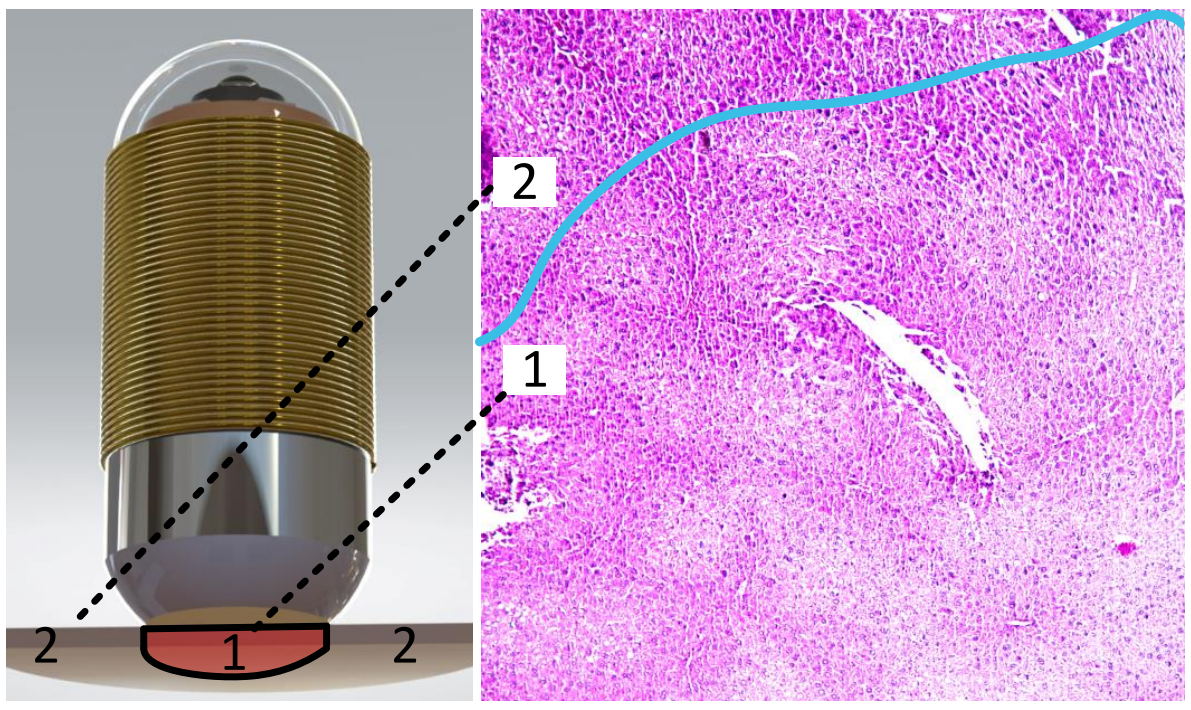

Fig. S6 Comparative analysis of thermally ablated regions. Region 1 corresponds to thermally ablated rat-liver tissue, whereas Region 2 represents non-ablated tissue outside the treatment zone.

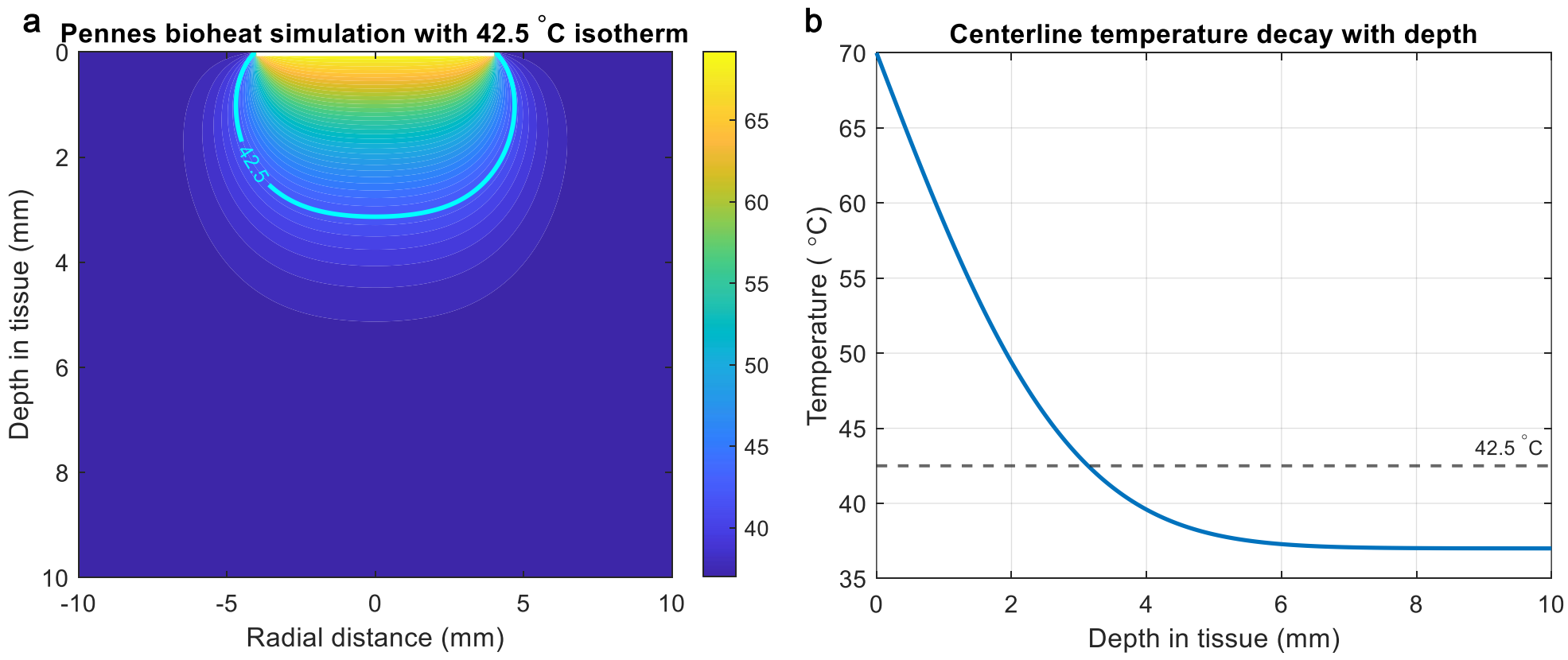

Fig. S7 Pennes bioheat simulation of thermal diffusion from the 8 mm-diameter capsule heating pad maintained at 70 °C in tissue initially at 37 °C. a, Steady-state temperature field showing the selected 42.5 °C reference isotherm (cyan). b, Central-axis temperature decay as a function of tissue depth.

The simulation provides only a preliminary theoretical estimation of thermal spread under the specified assumptions, suggesting that the elevated temperature may be concentrated near the heating pad. However, it does not constitute experimental validation of thermal safety. In particular, ablation depth, treatment margin, and injury to adjacent viable tissue have not yet been validated in perfused living tissue. Future in vivo studies will quantify these outcomes using real-time temperature monitoring, closed-loop power regulation, controlled heating durations, and post-treatment histological assessment. The treatment parameters will then be optimized to achieve effective thermal ablation while minimizing unnecessary injury to surrounding healthy tissue.

In addition to the local thermal-spread analysis around the ablation region, we evaluated the possible effect of whole-capsule heating on dynamic wireless-power reduction

and treatment time using a simplified bioheat model. Because the thermal experiments were conducted in ex vivo porcine stomach tissue, physiological heat-dissipation mechanisms, including blood perfusion and heat transfer through surrounding tissues and gastrointestinal fluids, were not fully reproduced. As shown in Fig. S8, the model predicts that an equivalent heat-generation power of approximately 4000 mW would be required for the whole-capsule temperature to approach the selected modeling reference temperature of 42.5 °C under the assumed in vivo conditions. In comparison, the estimated heat loss from the receiving coil and power electronics during WPT was below 1000 mW, corresponding to a predicted whole-capsule temperature increase of approximately 1.4 °C above the physiological baseline of 37 °C. Under these assumptions, the model predicts that substantial wireless-power reduction may not be required. However, these predictions are theoretical and require experimental validation in perfused living tissue; they should not be interpreted as an experimentally established thermal-safety threshold or treatment margin.

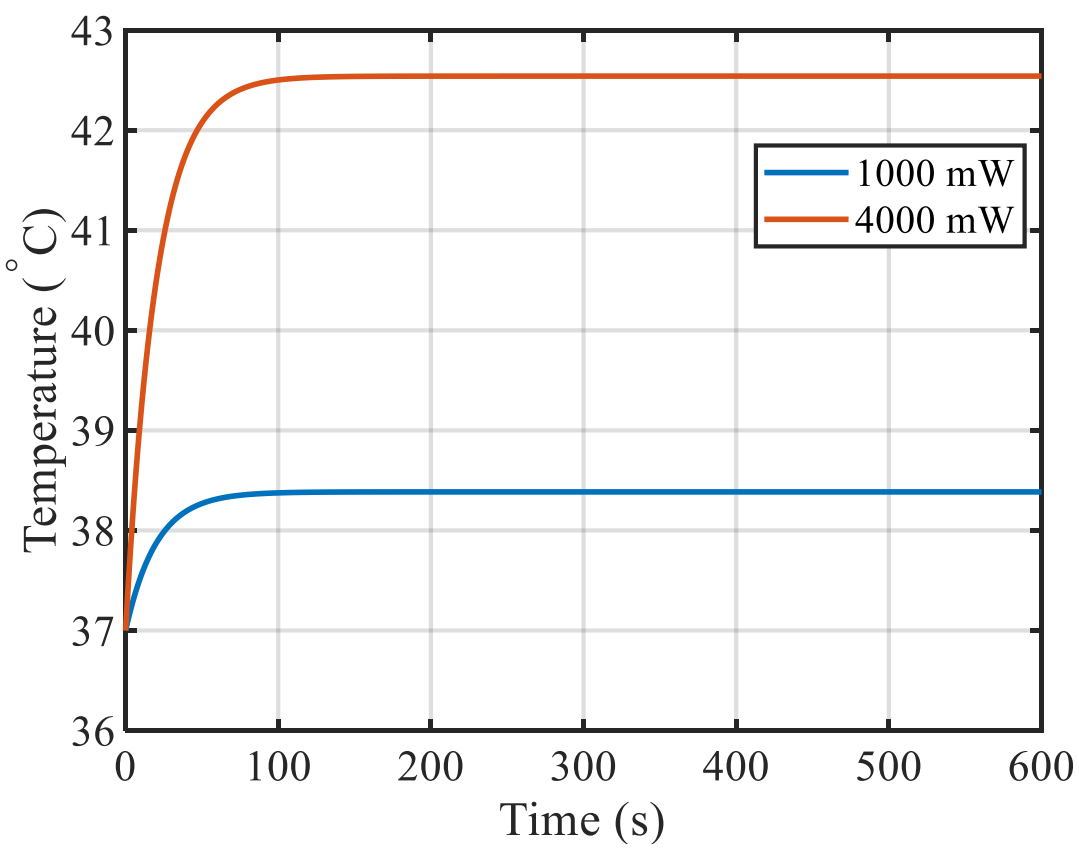

Fig. S8 Predicted whole-capsule temperature rise under different equivalent heat-generation powers based on the Pennes bioheat model.

The temperature decay of the MCR heating element in ambient air following thermal ablation is shown in Fig. S9. The workflow for histological analysis of tissue after thermal ablation is presented in Fig. S10.

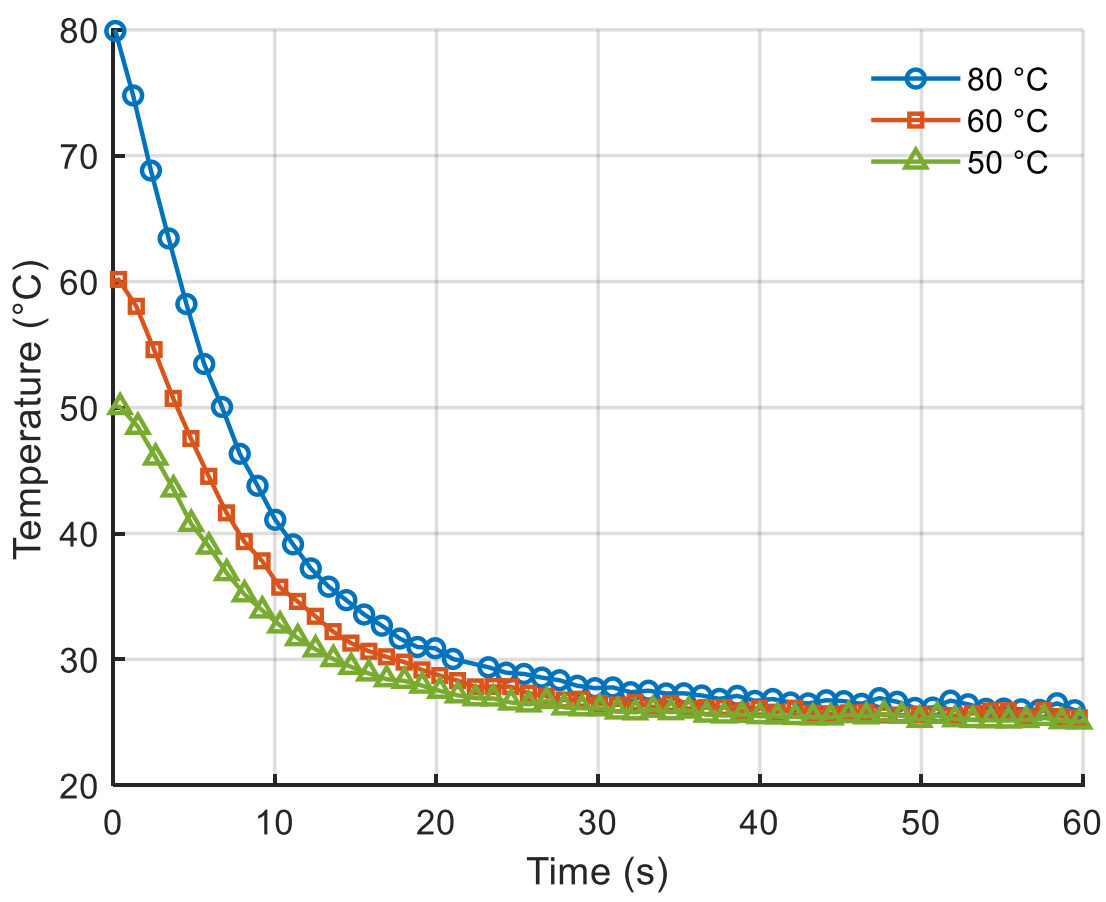

Fig. S9 Temperature decay of the MCR heating element in ambient air after thermal ablation, measured immediately after detachment from the target tissue.

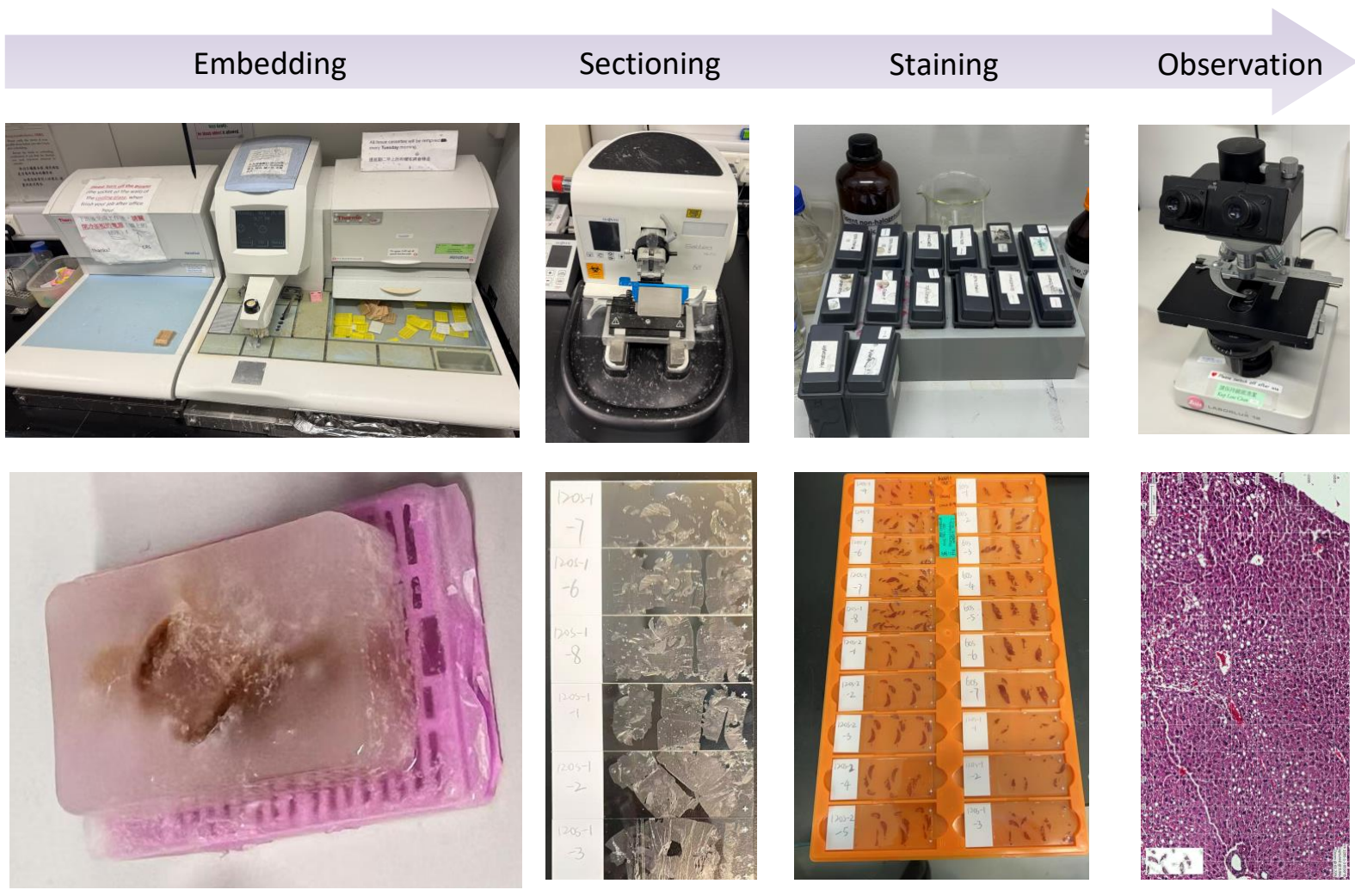

Fig. S10 Photos depict the complete workflow from treated tissue to histological assessment. The sequence shows the equipment and key steps, including tissue handling, paraffin embedding, microtome sectioning, slide preparation and staining, and microscopic examination of the final H&E-stained sections.

A.4 Magnetic field generation for WPT

The magnetic field $\boldsymbol{B}$ generated by the flexible transmitting coil during wireless charging can be calculated using the Biot-Savart law [2], as expressed by the following equation.

$$\boldsymbol{B} = \oint_L \mathrm{d}\boldsymbol{B} = \frac{\mu_0}{4\pi} \oint_L \frac{I\mathrm{d}\boldsymbol{l} \times \boldsymbol{r}'}{r'^3}. \tag{1}$$

where $\mu_0 = 4\pi \times 10^{-7}$.

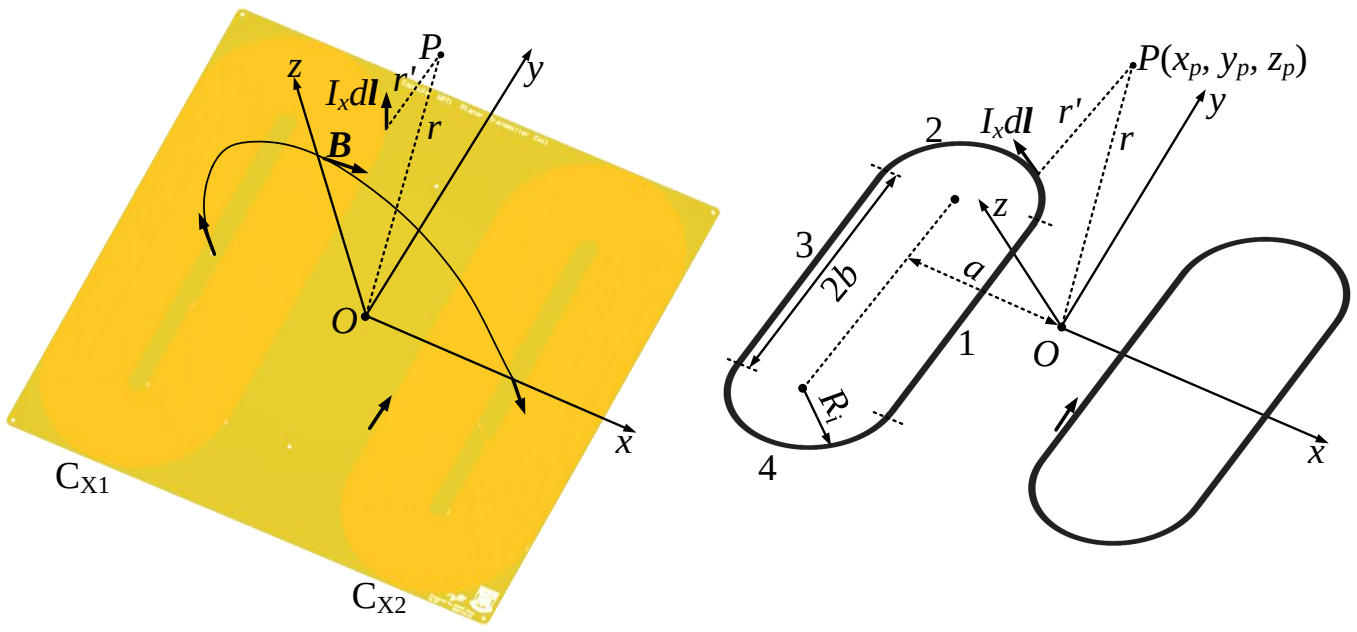

Fig. S11 Analysis of the magnetic field generated by coil C_{XX}.

As illustrated in Fig. S11, the magnetic field generated by coil C_{XX} at a spatial point P can be calculated using Eq. (1). Each circular loop in C_{X1}, indexed as the ith loop, is divided into four segments: two linear segments (labeled 1 and 3) and two semicircular arcs (labeled 2 and 4) with radius R_i. The magnetic field contribution from each of these four segments at point P is calculated independently, and their vector sum yields the total magnetic flux density $\boldsymbol{B}_{Li}$ produced by the ith loop of the left coil C_{X1} at P, where $\boldsymbol{B}_{Li} = (B_{Lix}, B_{Liy}, B_{Liz})$. The explicit expressions for each component are given in Eqs. (2), (3), and (4).

$$\begin{aligned} B_{Lix} = \frac{\mu_0 I_x}{4\pi}\Big(&\int_{-b}^{b} \frac{z_p dy}{\left[(x_p+a-R_i)^2+(y_p-y)^2+{z_p}^2\right]^{1.5}} \\ &+ \int_0^{\pi} \frac{R_i z_p \cos\alpha d\alpha}{\left[(x_p+a-R_i\cos\alpha)^2+(y_p-b-R_i\sin\alpha)^2+{z_p}^2\right]^{1.5}} \\ &+ \int_b^{-b} \frac{z_p dy}{\left[(x_p+a+R_i)^2+(y_p-y)^2+{z_p}^2\right]^{1.5}} \\ &+ \int_{\pi}^{2\pi} \frac{R_i z_p \cos\alpha d\alpha}{\left[(x_p+a-R_i\cos\alpha)^2+(y_p+b-R_i\sin\alpha)^2+{z_p}^2\right]^{1.5}}\Big) \end{aligned} \tag{2}$$

$$\begin{aligned} B_{Liy} = \frac{\mu_0 I_x}{4\pi}\Big(&\int_0^{\pi} \frac{R_i z_p \sin\alpha d\alpha}{\left[(x_p+a-R_i\cos\alpha)^2+(y_p-b-R_i\sin\alpha)^2+{z_p}^2\right]^{1.5}} \\ &+ \int_{\pi}^{2\pi} \frac{R_i z_p \sin\alpha d\alpha}{\left[(x_p+a-R_i\cos\alpha)^2+(y_p+b-R_i\sin\alpha)^2+{z_p}^2\right]^{1.5}}\Big) \end{aligned} \tag{3}$$

$$\begin{aligned} B_{Liz} = \frac{\mu_0 I_x}{4\pi}\Big(&\int_{-b}^{b} \frac{-(x_p+a-R_i)dy}{\left[(x_p+a-R_i)^2+(y_p-y)^2+{z_p}^2\right]^{1.5}} \\ &+ \int_0^{\pi} \frac{R_i[(x_p+a-R_i\cos\alpha)\cos\alpha+(y_p-b-R_i\sin\alpha)\sin\alpha]d\alpha}{\left[(x_p+a-R_i\cos\alpha)^2+(y_p-b-R_i\sin\alpha)^2+{z_p}^2\right]^{1.5}} \\ &+ \int_b^{-b} \frac{-(x_p+a+R_i)dy}{\left[(x_p+a+R_i)^2+(y_p-y)^2+{z_p}^2\right]^{1.5}} \\ &+ \int_{\pi}^{2\pi} \frac{R_i[(x_p+a-R_i\cos\alpha)\cos\alpha+(y_p+b-R_i\sin\alpha)\sin\alpha]d\alpha}{\left[(x_p+a-R_i\cos\alpha)^2+(y_p+b-R_i\sin\alpha)^2+{z_p}^2\right]^{1.5}}\Big) \end{aligned} \tag{4}$$

The calculation of the magnetic flux density $\boldsymbol{B}_{Ri}$ produced by the right coil C_{X2} at point P follows the same approach as that of the left coil C_{X1}. Therefore, the total magnetic flux density $\boldsymbol{B}_{CX}$ generated by the entire coil pair C_{XX} at point P can be expressed as:

$$\boldsymbol{B}_{CX} = \sum_{i=1}^{N_{CX}} (\boldsymbol{B}_{Li} + \boldsymbol{B}_{Ri}) \tag{5}$$

where N_{CX} denotes the total number of turns in either C_{X1} or C_{X2}, which are identical.

The derivation of the magnetic flux density $\boldsymbol{B}_{\mathrm{CY}}$ generated by the coil $\mathrm{C_{YY}}$ at point P follows the same procedure as that of $\mathrm{C_{XX}}$ and is therefore omitted here for brevity.

The magnetic flux density $\boldsymbol{B}_{Zk}$, expressed as $\boldsymbol{B}_{Zk} = (B_{Zkx}, B_{Zky}, B_{Zkz})$, generated by the kth turn of the coil $\mathrm{C_Z}$ with radius R_k at point P, is given by equations (6), (7), and (8).

$$B_{Zkx} = \frac{\mu_0 I_z}{4\pi} \int_0^{2\pi} \frac{R_k z_p \cos\alpha \mathrm{d}\alpha}{\left[(x_p - R_k\cos\alpha)^2 + (y_p - R_k\sin\alpha)^2 + {z_p}^2\right]^{1.5}} \tag{6}$$

$$B_{Zky} = \frac{\mu_0 I_z}{4\pi} \int_0^{2\pi} \frac{R_k z_p \sin\alpha d\alpha}{\left[(x_p - R_k\cos\alpha)^2 + (y_p - R_k\sin\alpha)^2 + {z_p}^2\right]^{1.5}} \tag{7}$$

$$\begin{aligned} B_{Zkz} = \frac{\mu_0 I_z}{4\pi} (&\int_0^{2\pi} \frac{R_k (x_p - R_k\cos\alpha)\cos\alpha d\alpha}{\left[(x_p - R_k\cos\alpha)^2 + (y_p - R_k\sin\alpha)^2 + {z_p}^2\right]^{1.5}} \\ + &\int_0^{2\pi} \frac{R_k (y_p - R_k\sin\alpha)\sin\alpha d\alpha}{\left[(x_p - R_k\cos\alpha)^2 + (y_p - R_k\sin\alpha)^2 + {z_p}^2\right]^{1.5}}) \end{aligned} \tag{8}$$

The total magnetic flux density generated by the coil $\mathrm{C_Z}$ at point P can be expressed as

$$\boldsymbol{B}_{\mathrm{CZ}} = \sum_{k=1}^{N_{\mathrm{CZ}}} \boldsymbol{B}_{Zk}. \tag{9}$$

where N_{CZ} denotes the total number of turns of the coil $\mathrm{C_Z}$.

Therefore, the magnetic flux density at point P generated by the multilayer planar transmitting coil can be expressed as

$$\boldsymbol{B}_P = \boldsymbol{B}_{\mathrm{CX}} + \boldsymbol{B}_{\mathrm{CY}} + \boldsymbol{B}_{\mathrm{CZ}}. \tag{10}$$

By applying the magnetic field model in equation (10), the charging power can be regulated through the control of the magnetic field intensity. The output voltage of the receiving coil is given by

$$U_{\mathrm{r}} = k_{\mathrm{r}} N_{\mathrm{r}} w \cos(\gamma) \left|\boldsymbol{B}_P\right| S, \tag{11}$$

where k_{r} is the mutual inductance enhancement factor introduced by the ferrite ring, S is the cross-sectional area of a single turn of the receiving coil, and γ denotes the angle between the magnetic field vector and the capsule's axial direction. In this work, γ is controlled to remain at or near zero, thereby maximizing power transfer efficiency.

The experimental setup for wireless charging performance testing is illustrated in Fig. S12.

A.5 Magnetic localization and actuation

The magnetic localization system is integrated into the base of the examination table and comprises 72 three-axis magnetometer sensors (Melexis MLX90393 Triaxis), arranged in two overlapping grids: a 7×6 grid with 60 mm spacing and an inner 6×5 grid offset by 30 mm. A custom magnetic field acquisition module, built around a an STM32F103C8T6 microcontroller (STMicroelectronics), collects data from all 72 sensors via $\mathrm{I^2C}$ communication.

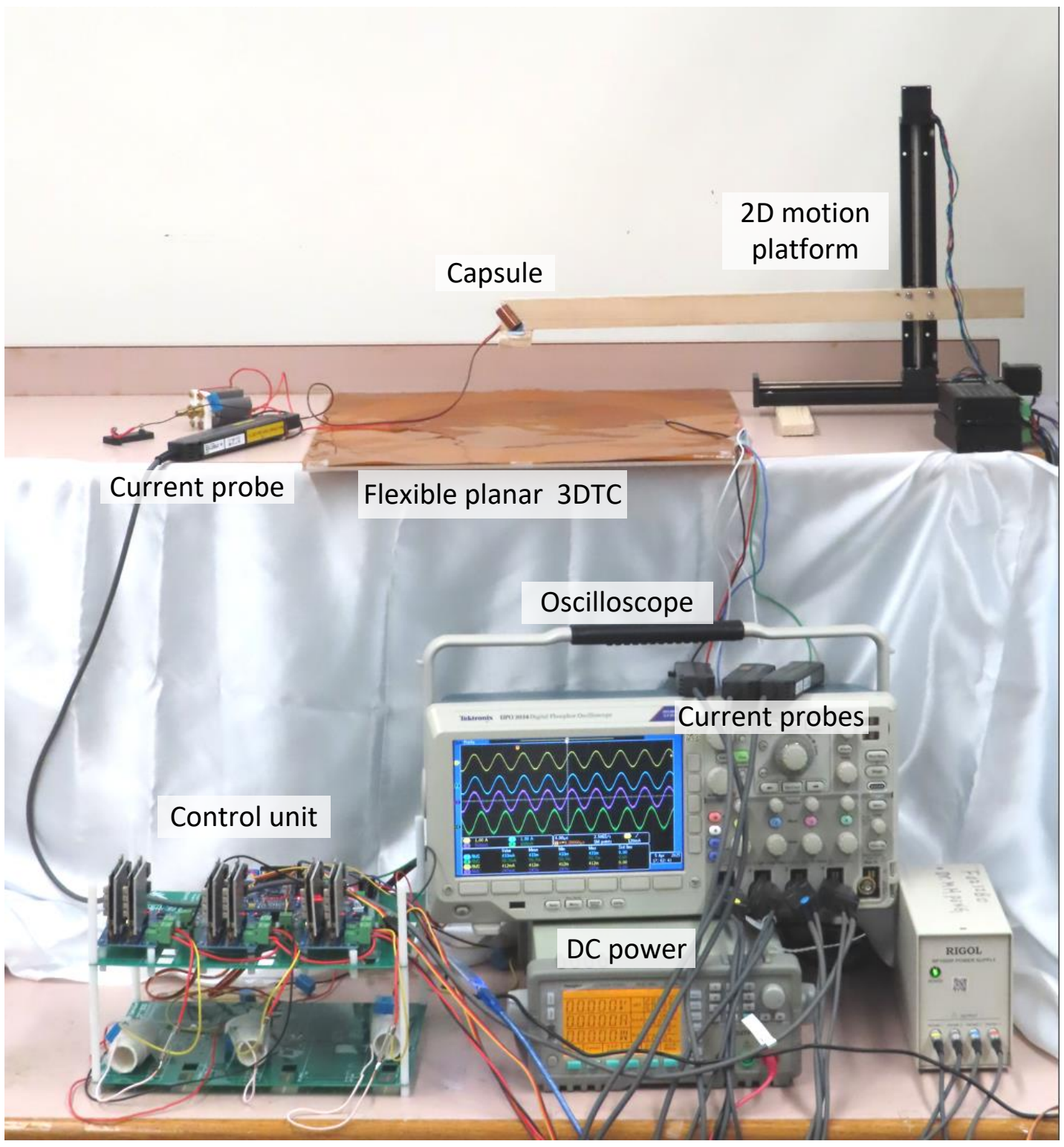

Fig. S12 Testing platform for the flexible planar wireless power transmitter.

The magnetic field generated by the magnet inside the MCR at the s-th sensor is modeled as a magnetic dipole and can be expressed by

$$\mathbf{B}(s) = \frac{\mu_0}{4\pi\|\mathbf{r}(s)\|^3}\left(3\hat{\mathbf{r}}(s)\hat{\mathbf{r}}(s)^\top - \mathbf{I}_3\right)\mathbf{m} \tag{12}$$

with $\hat{\mathbf{r}}(s) = \mathbf{r}(s)/\|\mathbf{r}(s)\|$, where μ_0 is magnetic permeability, $\mathbf{r}(s) = \mathbf{P}(s) - \mathbf{P}_c$ is the distance between measured sensor and magnet, and $\mathbf{m}$ is the magnetic moment for the magnet.

Let the magnetic field at the s-th sensor be $\mathbf{B}_a(s)$, which can be known by calculating the pose of robotic arm. Recalling the magnetic dipole relationship, it gives:

$$\mathbf{B}_c(s) = \mathbf{B}_s - \mathbf{B}_a(s) \tag{13}$$

where $\mathbf{B}_s$ is the measured magnetic field at the s-th sensor. Combining (12) and (13) gives the optimization function:

$$(\mathbf{P}_c, \mathbf{m}) = \arg\min_{\mathbf{P}_c,\mathbf{m}} \sum_{s=1}^{72} \|\mathbf{B}_c(s) - \mathbf{B}(s)\| \tag{14}$$

which provides the localization results at the position $\mathbf{P}_c$ and moment heading $\mathbf{m}$ of the magnet inside the MCR.

The magnetic actuation was implemented using two controllers. One controller regulated the MCR's position by generating the desired magnetic field $\mathbf{B}_a$, while the other controlled the MCR's heading to servo toward the target point.

A KUKA Med 7 robotic arm (KUKA AG) uses its joint variables $\mathbf{q}$ to position and orient an external permanent magnet (EPM). The robotic arm is connected to a personal computer (PC) via Ethernet communication. The geometric Jacobian of the KUKA Med 7 is denoted as $\mathbf{J}_{ro}$. The position and heading of the EPM are represented by $\mathbf{P}_E$ and $\mathbf{m}_E$, respectively. The magnetic force and torque acting on the MCR are obtained by

$$\begin{bmatrix} \delta\mathbf{F} \\ \delta\tau \end{bmatrix} = \mathbf{J}_{mag} \begin{bmatrix} \delta\mathbf{P}_E \\ \delta\hat{\mathbf{m}}_E \end{bmatrix} = \begin{bmatrix} \frac{\delta\mathbf{F}}{\delta\mathbf{P}_E} & \frac{\delta\mathbf{F}}{\delta\hat{\mathbf{m}}_E} \\ \frac{\delta\tau}{\delta\mathbf{P}_E} & \frac{\delta\tau}{\delta\hat{\mathbf{m}}_E} \end{bmatrix} \begin{bmatrix} \delta\mathbf{P}_E \\ \delta\hat{\mathbf{m}}_E \end{bmatrix} \tag{15}$$

where $\mathbf{J}_{mag}$ is the magnetic actuation Jacobian. The changes in $\delta\mathbf{F}$ and $\delta\tau$ leaded to the change of the position and heading of MCR, which is defined as

$$\begin{bmatrix} \delta\mathbf{F} \\ \delta\tau \end{bmatrix} = pid \begin{bmatrix} \mathbf{P}_{desired} - \mathbf{P}_c \\ \omega_{\text{des}} \end{bmatrix} \tag{16}$$

where *pid* refers to the proportional-integral-derivative design in position and heading control and $\mathbf{P}_{desired}$ is the control input. A position control is implemented to reach the target location.

The anchored MCR is then controlled using visual servoing to align with the target point based on the onboard camera images. The visual servo error is defined as

$$\tilde{\mathbf{e}} = R(\mathbf{m}_c) \begin{bmatrix} u - u^* \\ v - v^* \end{bmatrix}$$

where u and v are the desired pixel for the target point in camera view. u^* and v^* are assigned as the center pixel in camera view. $R(\mathbf{m}_c)$ is the rotation matrix derived form MCR heading $\mathbf{m}_c$ and IMU, which provide the rotation mapping from the world frame to the camera frame. The visual servo error is then used to compute the angular velocity of the camera as

$$\omega_{\text{des}} = -\lambda L_\omega^+ \tilde{\mathbf{e}} \tag{17}$$

where L_ω^+ is the pseudoinverse of interaction matrix with

$$L_\omega = \begin{bmatrix} \frac{uv}{f} & -\frac{f^2+u^2}{f} & v \\ \frac{f^2+v^2}{f} & -\frac{uv}{f} & -u \end{bmatrix}$$

and λ and f are the known designed parameter and focal length of the camera, respectively. The parameter ω_{des} from (17) contributes to the controller design presented in (16). Combining the magnetic force and torque model introduced in (15), the relationship between the joints and the desired $\delta\mathbf{F}$ and $\delta\tau$ is obtained as

$$\begin{bmatrix} \delta\mathbf{P}_E \\ \delta\hat{\mathbf{m}}_E \end{bmatrix} = \mathbf{J}_{r-m}\delta\mathbf{q} = \begin{bmatrix} \mathbb{I} & \mathbb{O} \\ \mathbb{O} & \mathbb{S}(\hat{\mathbf{m}}_E) \end{bmatrix} \mathbf{J}_{ro}\delta\mathbf{q}$$

and

$$\delta\mathbf{q} = \mathbf{J}_{r-m}^{+}\mathbf{J}_{mag}^{+}\begin{bmatrix}\delta\mathbf{F}\\ \delta\tau\end{bmatrix} = \mathbf{J}_{r-m}^{+}\mathbf{J}_{mag}^{+} \quad pid\begin{bmatrix}\mathbf{P}_{desired}-\mathbf{P}_{c}\\ -\lambda L_{\omega}^{+}\tilde{\mathbf{e}}\end{bmatrix} \tag{18}$$

where $\mathbb{I}$ and $\mathbb{O}$ are corresponding identity and zero matrix. $\mathbb{S}()$ and subscript $^{+}$ indicate the skew-symmetric form of the cross-product operation and pseudoinverse operation, respectively.

When $u, v \rightarrow u^{*}, v^{*}$ is satisfied, the current heading $\mathbf{m}_c^{\diamond}$ is used as the reference for constructing a magnetic potential-energy well, which is generated by an additional magnetic source producing the field $\mathbf{B}_w$. Therefore,

$$U = -\mathbf{m}_c^{\diamond}\mathbf{B}_w \tag{19}$$

the direction of $\mathbf{B}_w \longrightarrow -\mathbf{m}_c^{\diamond}$ and $\mathbf{B}_w$ increases along $\mathbf{m}_c^{\diamond}$, making an increase U in $\mathbf{m}_c^{\diamond}$ direction to achieve anchor in target point with reversed heading.

A.6 Circuit

The circuit schematic of the MCR is shown in Fig. S13.

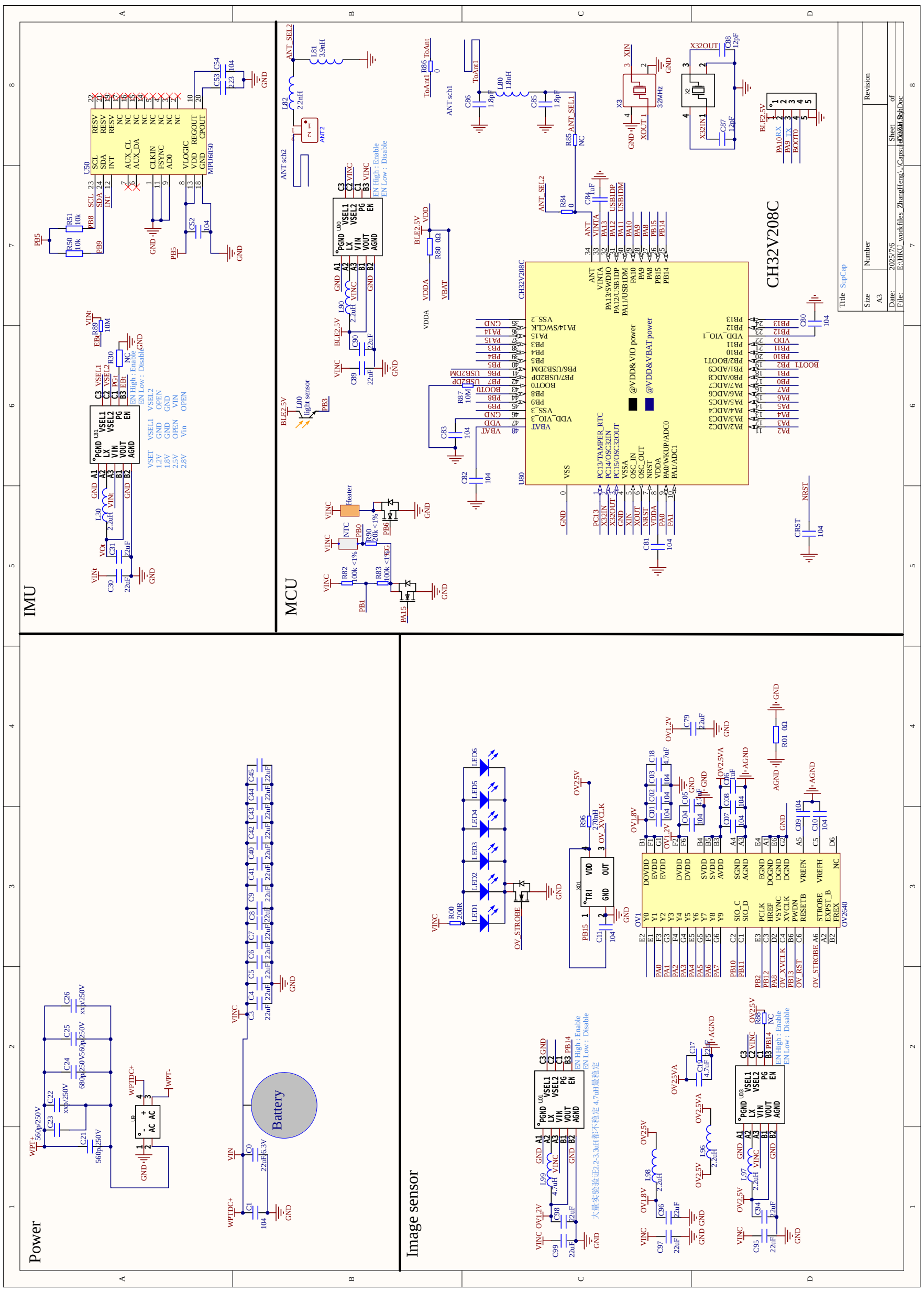

Fig. S13 Schematic diagram of the MCR.

References

[1] H. H. Pennes, "Analysis of Tissue and Arterial Blood Temperatures in the Resting Human Forearm," *Journal of applied physiology* 1, no. 2 (1948): 93–122.

[2] H. Kalhor, "Comparison of Ampere's Circuital Law (acl) and the Law of Biot-Savart (lbs)," *IEEE Transactions on Education* 31, no. 3 (1988): 236–238. 28
